# A synaptic learning rule for exploiting nonlinear dendritic computation

**DOI:** 10.1016/j.neuron.2021.09.044

**Published:** 2021-12-15

**Authors:** Brendan A. Bicknell, Michael Häusser

**Affiliations:** 1 Wolfson Institute for Biomedical Research, University College London, London WC1E 6BT, UK; 2 Department of Neuroscience, Physiology, and Pharmacology, University College London, London WC1E 6BT, UK

**Keywords:** dendritic computation, synaptic plasticity, pyramidal neuron, biophysical model, cable theory, morphology, NMDA receptors, learning rule, feature-binding problem

## Abstract

Information processing in the brain depends on the integration of synaptic input distributed throughout neuronal dendrites. Dendritic integration is a hierarchical process, proposed to be equivalent to integration by a multilayer network, potentially endowing single neurons with substantial computational power. However, whether neurons can learn to harness dendritic properties to realize this potential is unknown. Here, we develop a learning rule from dendritic cable theory and use it to investigate the processing capacity of a detailed pyramidal neuron model. We show that computations using spatial or temporal features of synaptic input patterns can be learned, and even synergistically combined, to solve a canonical nonlinear feature-binding problem. The voltage dependence of the learning rule drives coactive synapses to engage dendritic nonlinearities, whereas spike-timing dependence shapes the time course of subthreshold potentials. Dendritic input-output relationships can therefore be flexibly tuned through synaptic plasticity, allowing optimal implementation of nonlinear functions by single neurons.

## Highlights

• A learning rule derived from cable theory is used in biophysical simulations
• Pyramidal cell I/O functions can be optimized for computation by synaptic plasticity
• Active and passive dendritic mechanisms enhance input pattern discrimination
• Single neurons can learn network-level computations simply by tuning synaptic weights

## Graphical abstract

**Figure:**
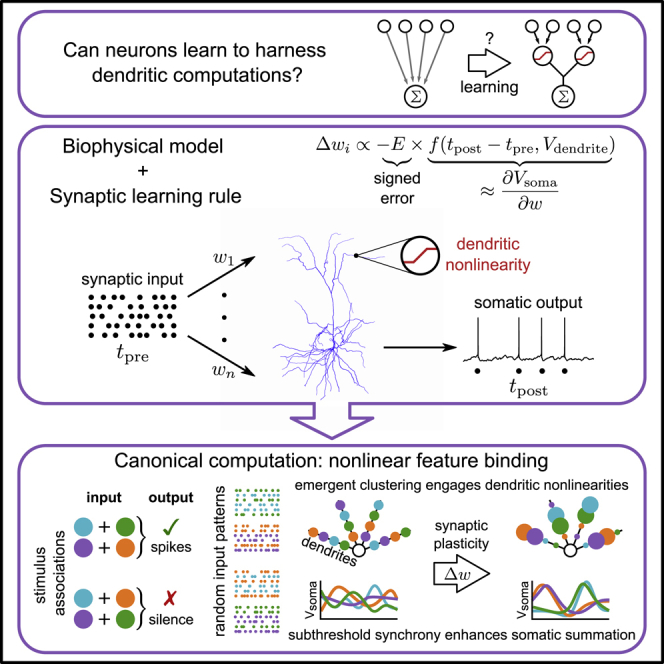

## Teaser

Bicknell and Häusser develop a theoretical approach for investigating single-neuron computation and learning. By deriving a plasticity rule that optimally adjusts the strengths of interacting synapses to control somatic spiking, they show that neurons can learn to harness the biophysical properties of their dendrites to perform nonlinear computations.

## Introduction

An essential role of each neuron in a circuit is to transform a barrage of synaptic input into a meaningful stream of action potential output. The majority of input to a neuron is received on the dendritic tree, potentially allowing transformations that extend far beyond the simple sum-and-threshold operation that is commonly assumed (London and Häusser, 2005; Silver, 2010; Poirazi and Papoutsi, 2020). Understanding the cellular basis of brain function therefore demands an understanding of how single-neuron input-output functions are constrained by physiology and how they can be tuned for processing information. Dendritic morphology controls the interactions between inputs distributed in space and time and determines the attenuation and shape of synaptic potentials propagated to the soma (Rall, 1967; Vetter et al., 2001; Williams and Stuart, 2002; Koch, 2002). Voltage-dependent conductances modulate these dynamics in a highly nonlinear manner (Stuart et al., 1997; Larkum et al., 1999; Magee, 1999; Schiller et al., 2000; Polsky et al., 2004; Nevian et al., 2007; Major et al., 2013), yielding modes of synaptic integration spanning sublinear to supralinear regimes (Cash and Yuste, 1999; Polsky et al., 2004; Tran-Van-Minh et al., 2015). In pyramidal neurons, it has been proposed that the combination of dendritic morphology and local NMDA receptor-dependent nonlinearities form a hierarchical processing structure with substantial computational power (Mel, 1992b; Archie and Mel, 2000; Poirazi and Mel, 2001). In the first stage of processing, synaptic input is integrated nonlinearly within individual dendrites, followed by a subsequent stage in which current flowing from dendrites is integrated at the soma, elegantly summarized as an equivalence of single neurons to multilayer neural networks (Poirazi et al., 2003a, 2003b; Jadi et al., 2014; Ujfalussy et al., 2018; Beniaguev et al., 2021; Jones and Kording, 2021a). However, this influential theory is incomplete, as it is yet to be comprehensively determined how or whether computations that capitalize on dendritic physiology can be learned.

Studies of dendritic processing have focused largely on how the responses of neurons differ when the rates, spatial distribution or timing of synaptic inputs are varied (London and Häusser, 2005; Silver, 2010). Computation in this context, such as selective responses to clustered input (Mel, 1992a; Cazé et al., 2013) or ordered sequences (Rall, 1964; Branco et al., 2010; Bhalla, 2017), is thus inherited from presynaptic firing patterns and connectivity. Although these computations may be accessible through precise programs of development or rearrangement of axonal connections (Mel, 1992a; Poirazi and Mel, 2001), a reliance on targeted wiring for exploiting dendritic mechanisms neglects synaptic plasticity as a dominant form of learning in the brain. Indeed, the concept of a neuron as a multilayer network instead evokes an image of a highly flexible device, whereby appropriate responses are learned through tuning of synaptic weights. Although many biological factors underlying plasticity are understood in molecular detail (Zucker and Regehr, 2002; Sjöström et al., 2008), at the computational level, the extent to which changes in synaptic strength can be harnessed to control the input-output function of a neuron is unknown.

Foundational theoretical studies have addressed this problem in simplified cell models, using supervised learning algorithms to investigate the limits of computation in single-compartment “point neurons” (Rosenblatt, 1958; Brunel et al., 2004; Legenstein et al., 2005; Gütig and Sompolinsky, 2006). More recent models that incorporate additional coarse-grained compartments to represent a dendritic tree have begun to reveal a variety of enhancements that may be accessible with a spatially extended morphology (Wu and Mel, 2009; Legenstein and Maass, 2011; O’Donnell and Sejnowski, 2014; Urbanczik and Senn, 2014; Schiess et al., 2016; Hawkins and Ahmad, 2016; Kastellakis et al., 2016; Guerguiev et al., 2017; Sacramento et al., 2018; Ujfalussy and Makara, 2020; Moldwin et al., 2021; Sezener et al., 2021; Jones and Kording, 2021a, 2021b). However, as model detail is often traded off against mathematical tractability in these approaches, it is possible that crucial features of neuronal function may have been lost, or artificially gained, by excluding important biological constraints. A realistic dendritic tree, for instance, presents not only opportunities for computation but also significant impediments that must be overcome (Häusser and Mel, 2003; Moldwin and Segev, 2020). Studies using detailed biophysical models have instead focused on exploring the consequences of experimentally motivated unsupervised rules (Bono and Clopath, 2017) or plasticity rules that implicitly assume only linear integration (Steuber et al., 2007; Moldwin and Segev, 2020), but whether these forms of learning realize the full potential of single neuron computation is unclear.

Here, we show that the computational power of dendritic processing can be flexibly exploited through synaptic plasticity. Although our approach is general, we focus on understanding how dendritic morphology and NMDA receptor-dependent excitability can be recruited without requiring structured connectivity. We analyze a detailed biophysical model of a layer 2/3 pyramidal neuron using a set of variational equations that reveal how the somatic membrane potential depends on the history of input to the dendritic tree. Using this to construct a local learning rule, we train the model to perform a nonlinear classification task. With rate-coded input, we find that synaptic weights evolve to selectively engage dendritic nonlinearities, consistent with the proposed equivalence to multilayer networks. We then extend this theory to encompass dendritic integration of temporal signals, revealing an effective spatiotemporal strategy for processing bursts of synaptic input. The trained models predict observable signatures of optimal processing by single pyramidal neurons, reflecting computations that are neither hand-tuned nor inherited but arise naturally when plasticity is governed by the constraints of physiology.

## Results

To understand the dendritic computations that can be learned through synaptic plasticity, we developed a compartmental model of a pyramidal neuron and a learning rule to tune its synaptic weights. The main technical contribution of this paper is that we show how a synaptic credit assignment problem can be addressed by using cable theory to account for complex nonlinear interactions in the dendritic tree and that this can be approximated at each synapse using only local signals. By training the model to perform a task, we aim to uncover the computational strategies accessible to single neurons, without presupposing the ultimate implementation. Specifically, after introducing the model and learning rule, we consider a nonlinear feature-binding problem similar to that studied by Legenstein and Maass (2011) and Cazé et al. (2013). This requires the model neuron to classify conjunctions of synaptic input patterns by producing spikes in response to a prescribed set of inputs but remaining silent for others. As this biologically motivated task demands nonlinear processing of synaptic input, it is ideal for examining the ability of single neurons to learn network-level computations.

### Synaptic integration in a layer 2/3 pyramidal neuron

**Figure 1:**
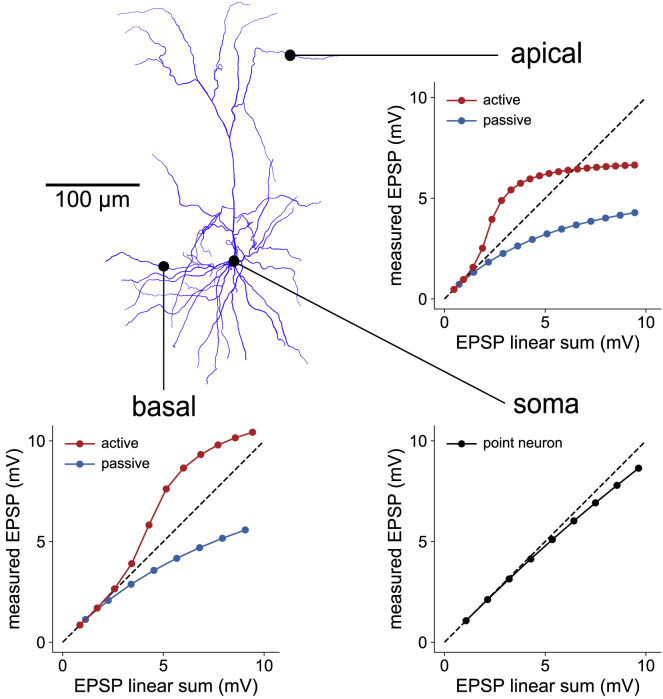
Synaptic integration depends on dendritic morphology and local nonlinearity Layer 2/3 pyramidal cell morphology with distinct basal and apical dendritic domains. Plots show the simulated peak somatic response to increasing numbers of excitatory synaptic inputs at the indicated locations, compared with the peak of the linear sum of the same number of unitary EPSPs. The basal and apical inputs are located at path distances of 95 and 275 μm from the soma, respectively. Voltage-dependent NMDA receptors yield supralinear integration within dendritic branches (active model, red lines), whereas integration in a purely passive model is sublinear (passive model, blue lines). Integration is approximately linear when the synapses of the active model are relocated to the soma (point neuron model, black line).

We constructed the model using a detailed morphology of a layer 2/3 pyramidal neuron from mouse primary visual cortex (Allen Institute for Brain Science, 2015; ID 502359001) and fitted a somatic spiking mechanism with characteristic linear f-I curve and adaptive firing rate (Pospischil et al., 2008). Synapses were randomly distributed across basal and apical dendrites, comprising 800 excitatory synapses with AMPA and voltage-dependent NMDA conductances and 200 inhibitory synapses with GABA conductances. The voltage dependence of the NMDA conductance results in supralinear integration of excitatory synaptic input to single dendritic branches, once a threshold level of input is reached (Figure 1). This well-described nonlinearity (Schiller et al., 2000; Polsky et al., 2004; Nevian et al., 2007; Branco et al., 2010; Branco and Häusser, 2011) is the foundation of the multilayer-network analogy, with the responses of individual dendrites resembling the activation functions of artificial network units (Mel, 1992b; Poirazi et al., 2003b; London and Häusser, 2005). We refer to the model with excitable NMDA synapses as the “active model,” as the response to synaptic input is dominated by the voltage dependence of the NMDA conductance. In a subset of simulations, we extend this model to consider a more excitable regime in which voltage-dependent intrinsic conductances are also present throughout the dendritic tree (Figure S2). To isolate the contributions of NMDA-dependent excitability and morphology, we also consider two alternative models: a passive model in which we omit the voltage dependence of the NMDA receptors and a “point neuron” model that is biophysically identical to the active model but has all synapses located at the soma. In qualitative contrast to the active model, the passive model integrates sublinearly within dendritic branches because the dominant local effect of depolarization due to synaptic input is a reduction in synaptic driving force. The point neuron model, whose synapses are not subject to the high local voltages experienced in dendrites (Spruston, 2008), integrates approximately linearly at the soma (Figure 1).

For a given pattern of input and synaptic weights w (defined as peak synaptic conductance), the dynamics of the models are described by discretized dendritic cable equations, coupled to equations for the dynamics of active somatic conductances:

(Equation 1) $$\dot{v}=f\left(t,v,m,h,n,p;w\right),$$

(Equation 2) $${\dot{x}}_{\mu }=g_{\mu }\left(v_{0},x_{\mu }\right),\;x_{\mu }=m,h,n,p.$$

In Equation 1, v denotes the vector of voltages for each segment of the morphology. The nonlinear function f describes the activation of synaptic conductances, generation of somatic spikes, and the flow of axial and transmembrane currents. The variables $x_{\mu }$ in Equation 2 represent Hodgkin-Huxley gating variables for the spiking mechanism (*m*, *h*: fast transient Na^+^; *n*: delayed rectifier K^+^; *p*: slow voltage-dependent ${\text{K}}^{+}$). The functions $g_{\mu }$ describe the gating kinetics, which depend on the somatic voltage $v_{0}$. We provide the full details of the model dynamics in STAR Methods, (Equation 8), (Equation 9), (Equation 10), (Equation 11), (Equation 12).

For efficient learning via synaptic plasticity, a fundamental question must be addressed: during a barrage of synaptic input, what is the influence of each synapse on the spiking output of a neuron? When the solution to this credit assignment problem is known, powerful learning strategies can be used in which synaptic weights are selectively modified relative to their ability to control the output. A compelling demonstration of this principle, based on an integrate-and-fire point neuron, is provided by the tempotron learning rule (Gütig and Sompolinsky, 2006, 2009; Urbanczik and Senn, 2009). In the tempotron, synapses are modified in proportion to the gradient of the membrane potential with respect to the weights, allowing the voltage to be efficiently pushed above or below spiking threshold as demanded by a given computational task. Although proven to be highly effective for learning in simple point neurons, to our knowledge, fuller extensions that are able to exploit the complex dynamics of dendritic integration have not yet been developed. With this goal in mind, we augment Equations 1 and 2 with a system of variational equations for credit assignment that computes gradients of the dynamically evolving voltage with respect to the synaptic weights.

For the weight of synapse *i*, the variational equations are given by

(Equation 3) $$\dot{\frac{\partial v}{\partial w_{i}}}=\frac{\partial f}{\partial w_{i}}+\frac{\partial f}{\partial v}\frac{\partial v}{\partial w_{i}}+\underset{\mu }{\sum }\frac{\partial f}{\partial x_{\mu }}\frac{\partial x_{\mu }}{\partial w_{i}},$$

(Equation 4) $$\frac{\dot{\partial x_{\mu }}}{\partial w_{i}}=\frac{\partial g_{\mu }}{\partial v_{0}}\frac{\partial v_{0}}{\partial w_{i}}+\frac{\partial g_{\mu }}{\partial x_{\mu }}\frac{\partial x_{\mu }}{\partial w_{i}},\;x_{\mu }=m,h,n,p.$$

Integrated numerically for each $w_{i}$, these equations track the dependence over time of the voltage throughout the morphology on the synaptic weights and thereby the relative contributions of each synapse within the population. In particular, we are interested in the dependence that underlies the spiking output at the soma, $\frac{\partial v_{0}\left(t\right)}{\partial w_{i}}$. When the specific identity of a synapse is not important, we suppress the index and express this general quantity as $\frac{\partial v_{\text{soma}}}{\partial w}$. Because the derivatives are propagated through the model dynamics, we capture the influence of dendritic morphology, interactions between excitatory and inhibitory synapses, dendritic NMDA spikes, and active somatic integration. We provide the full details of the variational equations in STAR Methods, (Equation 13), (Equation 14), (Equation 15), (Equation 16), (Equation 17).

**Figure 2:**
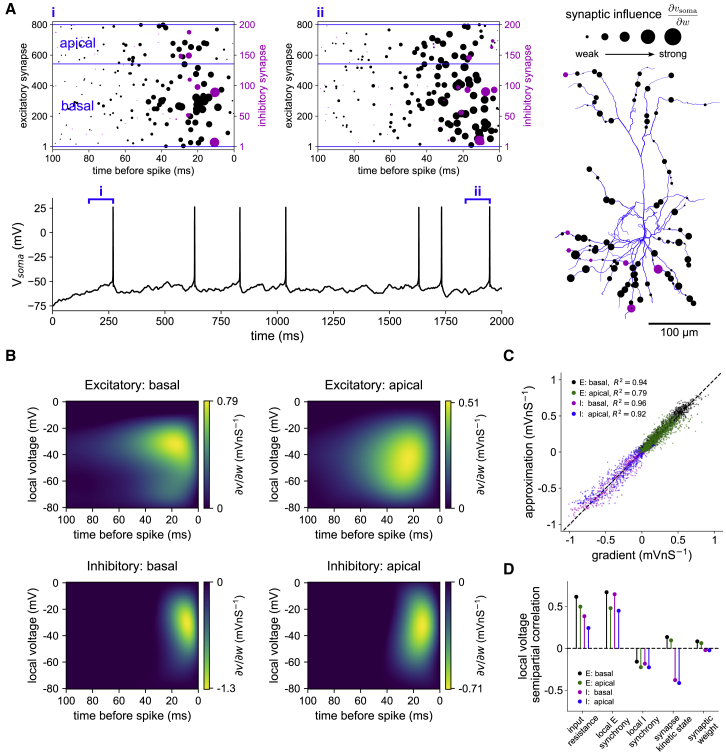
Two local variables determine the impact of synaptic plasticity on somatic output (A) Example simulation of the active model stimulated with Poisson input into excitatory (black) and inhibitory (magenta) synapses. Bottom left: somatic voltage trace. Top left: raster plots of synaptic input preceding two somatic spikes. Synapses located on the same dendrite are grouped together on the y axis. Markers are scaled by the magnitude of influence on the somatic voltage, $\frac{\partial v_{\text{soma}}}{\partial w}$, immediately prior to the spike, normalized by the maximum within excitatory and inhibitory groups. In this example, the variational equations were solved numerically for each individual synaptic activation by making dummy copies of synapses that were active more than once. Right: spatial distribution of activated synapses from example (ii). (B) Polynomial fits of somatic spike-triggered average $\frac{\partial v_{\text{soma}}}{\partial w}$ in the active model, to be used as plasticity kernels in the learning algorithm. (C) The approximations in (B) accurately predict the voltage gradients computed from numerical integration of (Equation 13), (Equation 14), (Equation 15), (Equation 16), (Equation 17) (fitted on 75% of the simulated data and tested on the remaining 25%). For visibility, scatterplot shows randomly sampled points from bins of 0.1 mV nS^−1^ width along the x axis (up to 100 points per bin). R^2^ values are computed from the correlation between actual and approximated values over all held-out data. (D) The voltage at a synapse at the time of somatic spikes depends on multiple factors, allowing their implicit representation in the learning rule. Shown is the semipartial correlation computed from a linear model fitted on 75% of the data and tested on the remaining 25%. See also Figure S1.

Using the numerical solution of Equations 3 and 4 to visualize $\frac{\partial v_{\text{soma}}}{\partial w}$ at times preceding action potentials, we find the influence of a synapse varies considerably with location, time of activation, and local interactions within the dendritic tree (Figure 2A). For instance, if excitatory synapses are active in spatial clusters, the impacts of weight changes are amplified because of voltage-dependent coupling of NMDA currents within the local branch (Figure 2A, example i). Potent inhibitory control is seen for inhibitory synapses that are proximal to active excitatory synapses, relative to the soma (Figure 2A, example ii, morphology lower right), and also on distal dendritic tips (Figure 2A, example ii, morphology upper left), in agreement with previous work (Gidon and Segev, 2012). By extending the standard compartmental equations to track internal model dynamics, we thus access an explicit readout of the interactions between input statistics and dendritic biophysics, and the influence of every synapse on the soma.

### Local variables can assign synaptic credit for somatic spikes

Equations 3 and 4 provide a general means to solve the problem of synaptic credit assignment in single neurons, without sacrificing the rigorous constraints and predictive power of biophysical simulations. However, there are two limitations to constructing a learning rule directly from this system. First, the inherent complexity means that numerical solution over potentially many training iterations is computationally expensive. Second, the equations are non-local, as the computed influence of a synapse formally depends on activity in even very distant regions of the dendritic tree, information unlikely to be generally available to synapses of a real neuron. To overcome these issues and gain further insight into the underlying principles, we sought an approximation using variables local to each synapse.

To construct the approximation, we performed an extensive set of simulations in which input rates, weights, and synaptic locations were varied. Pooling over synapses in either basal or apical dendrites, we computed a postsynaptic spike-triggered average of $\frac{\partial v_{\text{soma}}}{\partial w}$ as a function of the time that a given synapse was activated before the postsynaptic spike, and the local dendritic voltage at the time of the postsynaptic spike. These functions could be well described by polynomial fits (Figure 2B, active model shown). Approximating values of $\frac{\partial v_{\text{soma}}}{\partial w}$ at the time of postsynaptic spikes using the fitted spike-triggered averages accounted for ∼90% of the variance of held-out data (Figure 2C). The temporal components of the fits resemble time-reversed and cable-filtered synaptic conductances; the window of excitatory integration reflects a mix of AMPA and NMDA kinetics, and the peak influence of apical synapses is shifted to earlier activation times and reduced in magnitude relative to basal synapses (Figures S1A–S1C). In the active model, the peaks in the voltage components for both excitatory and inhibitory synapses coincide with the voltage-dependent activation of NMDA receptors between ∼−30 and −40 mV, revealing a small time and voltage window in which synaptic weight changes are maximally effective at modifying somatic output. The results for the passive model are similar, but with the peak in the voltage components for the excitatory synapses shifted to the somatic spiking threshold of ∼−50 mV (Figure S1D). We approximated $\frac{\partial v_{\text{soma}}}{\partial w}$ for the point neuron using only synaptic activation time, as in this case the local voltage is the somatic membrane potential itself (Figure S1C).

The output of Equations 3 and 4 can be accurately captured in terms of just two local variables, synaptic activation time and dendritic voltage, which is surprising given the complex morphology and impact of features such as spatiotemporal clustering of inputs (Figure 2A). This important sensitivity is conferred through the local voltage dependence, as we find that such features are represented indirectly through the voltage signal within each branch. We used linear regression to quantify the unique contributions of key structural and dynamic variables to the local voltage by their semipartial correlation (i.e., ability to predict the residual voltage after removing components explained by other variables). At the time of somatic spikes, the voltage at a synapse depends strongly on input resistance (related to its finer-scale location), and the degree of synchronous activation of other synapses on the same branch (within a 100 ms window) (Figure 2D). The voltage depends comparatively weakly on the weight or kinetic state (value of the temporal component of conductance) of a synapse itself, once other variables are accounted for (Figure 2D). This implies that sets of both excitatory and inhibitory synapses can be strongly coupled by the voltage within branches, and in principle, voltage-dependent plasticity can thereby act with sensitivity to the spatiotemporal structure of the input. Learning controlled by local variables can therefore harness the morphology and dynamics of a pyramidal neuron without an explicit representation of either.

We use these results to construct a supervised synaptic learning rule, inspired by the tempotron (Gütig and Sompolinsky, 2006), that accounts for nonlinear processing in the dendritic tree. In the following, we consider an application where a neuron should produce somatic spikes on presentation of noisy input patterns from a preferred class (+) but remain silent during presentation of patterns from a nonpreferred class (−). The goal of learning is to minimize the expected number of incorrect classifications across the full set of input patterns.

We use a greedy algorithm in which input patterns are presented sequentially and synaptic plasticity acts to either suppress or reinforce somatic spiking activity. We assume that a supervisory system maintains a running average of the classification error for each input pattern and uses this to guide the learning process. On presentation of (+) patterns, the supervisor encourages spiking by depolarizing the soma. On both (+) and (−) patterns, the supervisor controls the sign and globally scales the magnitude of plasticity, such as via a neuromodulator (Seol et al., 2007). Synaptic weights are modified whenever a somatic action potential is fired, which we assume is signaled throughout the dendrites by action potential backpropagation (Stuart and Sakmann, 1994; Stuart et al., 1997). We use the local approximations of $\frac{\partial v_{\text{soma}}}{\partial w}$ as plasticity kernels that assign the specific weight change for each synapse as a function of synaptic activation time and local dendritic voltage. Altogether, the weight update rule is given by

(Equation 5) $$\text{Δ}w_{i}=-\alpha \bar{E_{p}}\underset{k}{\sum }K_{i}\left(\text{Δ}t_{i}^{k},v_{{\text{dend}}_{i}}\right).$$

The first factor in Equation 5 is composed of a learning rate α and a running average error $\bar{E_{p}}$ provided by the supervisory system ($\bar{E_{p}}\leq 0$ for (+) patterns and $\bar{E_{p}}\geq 0$ for (−) patterns). This term adaptively modulates the magnitude of plasticity, such that weight updates are largest when the neuron has been performing poorly, and learning ceases once the noisy input patterns can be classified without error. In Figure S5 we show that this term can also be computed online from the spiking output of the neuron, in which case the supervisor need only provide a binary classification label. The second factor in Equation 5 acts locally to assign synaptic credit for the somatic spike; $K_{i}$ denotes the appropriate plasticity kernel for synapse *i* (basal, apical; excitatory, inhibitory), $\text{Δ}t_{k}$ is the time of the *k*th synaptic input relative to the somatic spike, and $v_{{\text{dend}}_{i}}$ is the local dendritic voltage at the time of the somatic spike. Thus, for example, an excitatory synapse in the basal dendrites that was activated 40 ms before a somatic spike, and subject to a local voltage of −40 mV at the time of the somatic spike, would be updated in proportion to the value read from the top left panel of Figure 2B: $\text{Δ}w_{i}\propto 0.44\;\text{nS}$, after unit conversions. Because the plasticity kernels approximate the gradient of somatic voltage with respect to the weights, synapses are modified in proportion to their relative ability to reinforce or suppress somatic spikes.

### Can a single neuron learn nonlinear functions?

**Figure 3:**
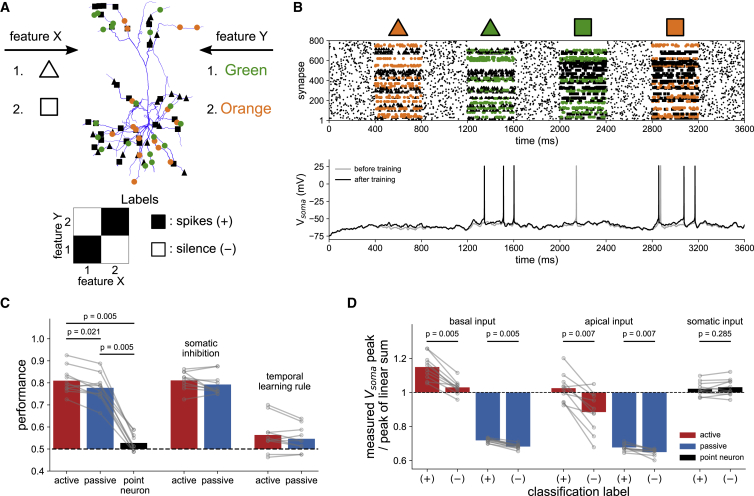
A single neuron can learn nonlinear functions (A) Nonlinear feature-binding problem. Synapses representing different stimulus features were randomly distributed throughout basal and apical dendrites. In this example, the neuron should only spike in response to the associations “green triangle” and “orange square” as indicated by the classification labels (bottom). (B) Example simulations of a model before (gray) and after (black) training on the task defined in (A). Each combination of features is presented in turn via rate-coded Poisson input, interspersed with background noise. For clarity, only input to excitatory synapses is shown. (C) Performance (fraction correct) of models trained on ten random instantiations of the task (left bars). In the somatic inhibition condition (middle bars), models were trained with all inhibitory synapses placed at the soma. Performance collapsed when dendritic voltage dependence was omitted from the learning rule (right bars). (D) Classification of associations is made by differential supralinear or sublinear integration. Input was presented to the indicated domains of trained models with somatic spiking blocked. The peak somatic depolarization measured when features were presented together was compared with the sum of responses when presented independently (averaged over 20 presentations of each association pair, then over label types). All bars denote means; p values are from two-tailed Wilcoxon signed-rank tests between groups for n = 10 independent replications. See also Figures S2 and S3.

We challenged the active, passive, and point neuron models with a nonlinear feature-binding task, framed as binary classification of combinations of synaptic input patterns (Legenstein and Maass, 2011; Cazé et al., 2013; Tran-Van-Minh et al., 2015). This task is an abstraction of the tendency of cortical neurons to respond to selective conjunctions of variables, such as spatial orientation and frequency, or multi-modal stimuli (Leventhal et al., 1995). We first focused on the rate-coded input regime on which previous theories of pyramidal cell computation are based (Poirazi et al., 2003b; Jadi et al., 2014) and chose classification labels such that the task could not be solved by linear integration (Legenstein and Maass, 2011). We consider streams of input from two presynaptic populations representing different classes of stimulus features, for concreteness, shape, and color. The neuron must learn to spike on specific associations of features from each class, such as “green triangle” and “orange square,” but no other combinations, such as “green square,” even though similar groups of synapses are active on both preferred and nonpreferred associations (Figure 3A). Note that this task is similar to learning an exclusive OR (XOR), as historically studied in a machine learning context (Minsky and Papert, 2017), but differs by requiring selective responses to combinations of different input patterns, rather than input magnitudes. Biologically, this corresponds to the presynaptic populations encoding stimulus identity through patterns of activity rather than the binary presence or absence of stimuli as in the XOR. Previously proposed solutions to this problem require strong assumptions about plasticity of the coupling of dendritic branches to the soma (Legenstein and Maass, 2011), motivated by observations in hippocampal neurons (Losonczy et al., 2008), or structured connectivity that organizes specific inputs into clusters (Cazé et al., 2013; Tran-Van-Minh et al., 2015). Here, we neither assume a biophysical implementation nor hard-wire a solution through connectivity but let synaptic plasticity act on randomly distributed inputs to learn the task.

To map the task to patterns of synaptic input, we defined each feature component by a sparse, randomly generated vector of input rates taking values of either 0 or 40 Hz (see STAR Methods, (Equation 18), (Equation 19), (Equation 20)). Input patterns were realized in simulations as Poisson spike trains comprising an initial 100 ms of background activity to randomize the initial conditions, followed by a 400 ms “stimulus presentation” of pairs of features. We used a sparseness of 1/16 (fraction of active synapses) to construct the rate vectors, giving an average of 1,000 presynaptic spikes per stimulus presentation (i.e., one spike per synapse, facilitating later comparison with candidate temporal coding schemes). We confirmed that our results do not depend qualitatively on these specific parameter choices, which are comparable with related work (Poirazi et al., 2003b; Legenstein and Maass, 2011). Pairs of features were presented in random order over 1,000 epochs of training and then tested 20 times without input from the supervisor. After training, the active model could robustly discriminate preferred associations from nonpreferred associations or background noise, demonstrating the ability of our learning rule to tune the synaptic weights of a detailed biophysical model for nonlinear computation (Figure 3B).

Which mechanisms are recruited to solve this task? Comparing classification performance across the three models confirms the advantage of dendritic morphology, with the point neuron performing at chance levels (Figure 3C; active, 81% ± 6%; passive, 78% ± 6%; point neuron, 53% ± 4%; mean ± SD). Dendritic excitability, however, is not essential, as the passive model performed almost as well as the active model. The performance of both models remained high when trained with all inhibitory synapses placed at the soma (Figure 3C; active, 81% ± 4%; passive, 79% ± 5%; mean ± SD), pointing to the engagement of predominantly excitatory nonlinearities. The voltage dependence of the learning rule was crucial for recruiting these nonlinearities, as performance collapsed when restricting it to depend only on synaptic activation time (Figure 3C; active, 56% ± 7%; passive, 55% ± 5%; mean ± SD).

To explore the possible roles of supralinear and sublinear modes of integration, we simulated the response of the trained models to different feature components, individually and in pairs, while blocking somatic spiking. Summation of pairs of features was supralinear in basal dendrites of the active model for all associations, but substantially more so for the preferred class, relative to nonpreferred (Figure 3D). By contrast, summation in passive basal dendrites was sublinear, with a small but consistent bias toward stronger sublinearity on nonpreferred associations, and similarly for passive apical dendrites. Unexpectedly, apical dendrites of the active model also exhibited a pronounced sublinear response, reflecting a level of depolarization such that the saturating phase of the sigmoid nonlinearity was engaged. We found similar results when the model was extended to include dendritic Na^+^, K^+^, and HCN channels to increase the intrinsic excitability of the dendritic tree (Figure S2). Therefore, in agreement with the central premise of Poirazi et al. (2003b), selective engagement of active mechanisms in basal dendrites can indeed be harnessed for nonlinear computation. However, voltage-dependent plasticity can also recruit sublinear forms of processing, and with this flexibility, the optimal apical strategy predicted for this morphology in fact resembles passive integration.

**Figure 4:**
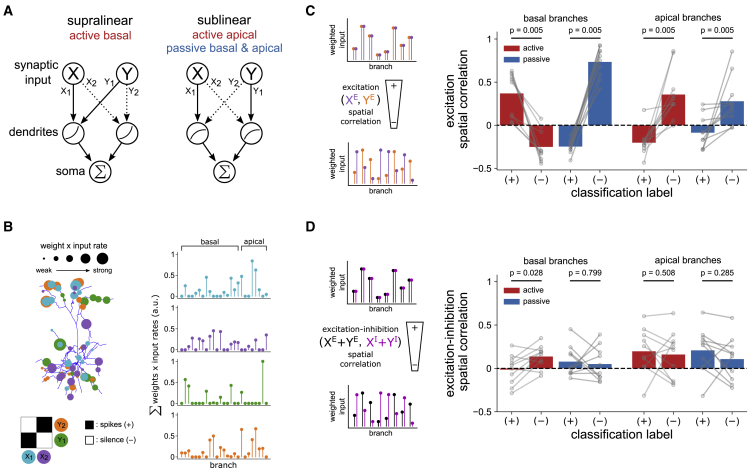
Learning tunes the spatial distribution of input strengths (A) Schematic illustrating how the feature-binding task can be solved via the structured connectivity mechanism proposed by Cazé et al. (2013). Arrows denote the targeting of excitatory input from a given feature to a compartment. With supralinear integration, responses to clustered input are enhanced relative to responses to dispersed input, and conversely with sublinear integration. (B) When connectivity is random, the strategies of (A) can be realized functionally by tuning synaptic weights through learning. Left: spatial distribution of excitatory input strengths (weight × input rate) in a trained model. Inputs are color-coded by the features they represent and the classification labels defined by the matrix below. Right: profiles of excitatory input strength for the model depicted on the left. The height of each point is proportional to the sum of weighted input rates on a branch. Preferred associations (e.g., $X_{1}$ and $Y_{1}$; blue and green) have strong inputs to common basal dendrites but separate apical dendrites. Conversely, strong inputs of nonpreferred associations (e.g., $X_{1}$ and $Y_{2}$; blue and orange) are dispersed in basal dendrites and clustered in apical dendrites. (C) Left: functionally clustered or dispersed configurations are reflected in the spatial correlation between weighted input to dendritic branches. $X^{\text{E}}$ and $Y^{\text{E}}$ represent excitatory input from two features. Right: correlation between spatial profiles of excitation (weighted input rates, summed within branches) from association pairs after learning. (D) As in (C) but comparing excitation and inhibition. In this case, the excitatory and inhibitory contributions from both features are summed before computing the correlation. In basal dendrites, spatially selective inhibition serves to suppress the response to (−) patterns. All bars denote means; p values are from two-tailed Wilcoxon signed-rank tests between groups for n = 10 independent replications.

Examining the weights of the trained models, we find that the learning rule rediscovers the clustering principles proposed in terms of structured connectivity in the binary neuron model of Cazé et al. (2013). Cazé et al. (2013) proposed that with an expansive nonlinearity, excitatory inputs from two features forming a preferred association should be spatially clustered to enable supralinear integration. Inputs from two features forming a nonpreferred association should instead be dispersed, thereby ensuring only linear integration and a smaller somatic response (Figure 4A, left). Conversely, with a suppressive nonlinearity, inputs forming preferred associations should be dispersed, with linear integration yielding a larger somatic response than the sublinear integration of clustered inputs (Figure 4A, right). Translated to our setting, in which connectivity is random, we find these strategies can be realized functionally through learning by tuning the spatial distribution of input strength (Figure 4B). To quantify this, we constructed a spatial profile of excitation strength for each feature in an association pair by summing the weighted excitatory input rates on each dendritic branch and then computed the correlation between profiles. Comparing profiles in basal and apical domains separately, differences in functional clustering, as defined by the overlap in patterns of excitation strength, reflect the pattern of supra- or sublinear summation observed in the simulations (Figure 4C). Comparing profiles of excitation and inhibition in a similar manner, we find that they overlap selectively in basal dendrites of the active model on nonpreferred associations, serving to further suppress the response to those association pairs (Figure 4D).

Although the performance of the active and passive models was surprisingly similar, there are several notable differences. Although the degree of functional clustering was comparable in both cases, this was translated into much larger differences in nonlinear summation in the active model (Figure 3D). This effect may be due to the presence of steeper dendritic nonlinearities in the active case (Figure 1), allowing a larger response difference between classes. Moreover, we find that the active model is more robust to input noise, tested by varying the rates of stimulus-dependent and background synaptic activity and the total number of synapses (Figures S3A–S3C). We also performed additional simulations in which the connectivity was highly structured, such that the solutions learned with random synapse placement in Figure 3 were either increasingly “hard-wired” or prohibited. Strikingly, in the latter case, whereas the passive model failed to learn the task, the active model learned to use an alternative strategy of sublinear processing in basal dendrites (Figures S3D and S3E). Active dendritic processing therefore enables more robust and flexible solution of the task. Generally, however, our results demonstrate that the power of multilayer integration is broadly accessible to single neurons through active and passive dendritic mechanisms, even with random connectivity. Spatial processing of rate-coded input can be learned in either case through selective enhancement of co-localized synaptic weights.

### Computation with temporal signals

We have shown how a single pyramidal neuron can learn to compute nonlinear functions of input rates. A long-standing question is whether analogous computational principles govern temporal processing within a branched dendritic tree (Poirazi et al., 2003b; London and Häusser, 2005; Jadi et al., 2014). One challenge in addressing this question is choosing a form of temporal input, as there are many possibilities for encoding information in spike timing, and the organization of *in vivo* synaptic input is unknown. We therefore proceed in two stages. Using the biophysical model as a guiding constraint, we first identify a form of input that maximizes performance on the feature-binding task, while respecting the stochasticity of *in vivo* spike generation and transmission. We then explore this regime as a hypothesis of temporal coding that is optimally suited to single neuron computation.

**Figure 5:**
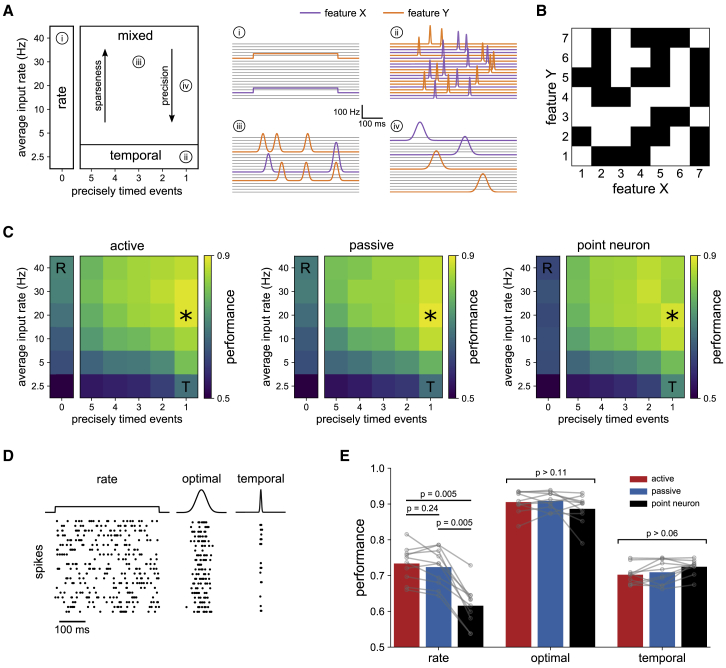
Sparse, precisely timed bursts of input maximize classification performance (A) Left: parameterization of candidate rate and temporal coding schemes by the time-averaged input rate to active synapses and the number of precisely timed elevations of the input rate (implemented as Gaussian bumps). The total presynaptic population rate is constrained to be the same for all parameters. To enforce this constraint, and with scaling to maintain physiological instantaneous input rates, patterns also differ in sparseness (fraction of active synapses) and temporal precision (width of rate elevations). Note that we use a decreasing order for the x axis in the temporal region; having multiple precisely timed events per synapse more closely resembles a rate code than a single event per synapse as the input spikes are more uniformly distributed in time. Right: example rate functions generated for an association pair in the feature-binding task by different parameter choices. (i) sparse rate code, (ii) dense temporal code, (iii) and (iv) mixed regimes comprising bursts of activity. For clarity, only 20 synapses are shown. (B) Example 7 × 7 matrix of associations to be classified. Classification labels are randomly assigned for each replication. (C) Performance (fraction correct) of trained active, passive, and point neuron models, averaged over 10 replications for each input condition. The optimal form of input (asterisk) was the same for all models. R and T denote the rate and temporal schemes used for comparison in (D) and (E). (D) Example realizations of Poisson input to a synapse for the rate, optimal, and temporal conditions. Bursts in the optimal condition are temporally localized but do not suffer from the transmission failures of the temporal condition. (E) Detailed comparison of performance across the three models from an independent set of simulations. Bars denote means; p values are from two-tailed Wilcoxon signed-rank tests between groups for n = 10 independent replications.

Extending the approach described above, we represented input features by randomly generated patterns of time-dependent presynaptic rates. We parameterized a broad space of candidate input schemes by the time-averaged firing rate of active synapses and the number of precisely timed events per active synapse (implemented as Gaussian bumps of elevated activity), while holding the total population rate constant (Figure 5A). This parameterization defines a space of possible input schemes ranging from a sparse rate code (as implemented in Figure 3) to a dense temporal code in which every synapse receives one presynaptic spike on average within a precise window of time (similar to Gütig and Sompolinsky, 2006, but here with stochastic spike generation). Interpolating between these extremes is a mixed regime in which inputs carry both rate and temporal information, communicated through single or multiple bumps of activity of varying size.

Using the same learning rule and training procedure as before, we found the introduction of temporal signals resulted in near-perfect performance on the 2 × 2 association task of Figure 3 (not shown). We therefore increased the computational load by extending the feature-binding task to classification of 7 × 7 randomly labeled associations (Figure 5B). We trained each of the three models on the 7 × 7 task using input patterns generated under the various parameterized input regimes. We find that the form of input that maximizes classification performance is the same for all models (Figure 5C). In the optimal input regime, sparsely activated synapses communicate in single ∼50 ms bursts, each comprising an expected eight presynaptic spikes (Figure 5D). Notably, across all models, synaptic input of this form resulted in a pronounced performance enhancement over the more commonly assumed purely rate or temporal schemes, as assessed with an independent set of simulations (Figure 5E; active, 73% ± 5%/90% ± 3%/70% ± 3%; passive, 72% ± 5%/91% ± 2%/71% ± 5%; point neuron, 62% ± 5%/89% ± 4%/72% ± 2%; mean ± SD for rate/optimal/temporal input). This advantage was observed for computational loads tested up to 10 × 10 associations and was robust to background noise and trial-by-trial perturbations to synaptic weights and burst timing (Figure S4).

**Figure 6:**
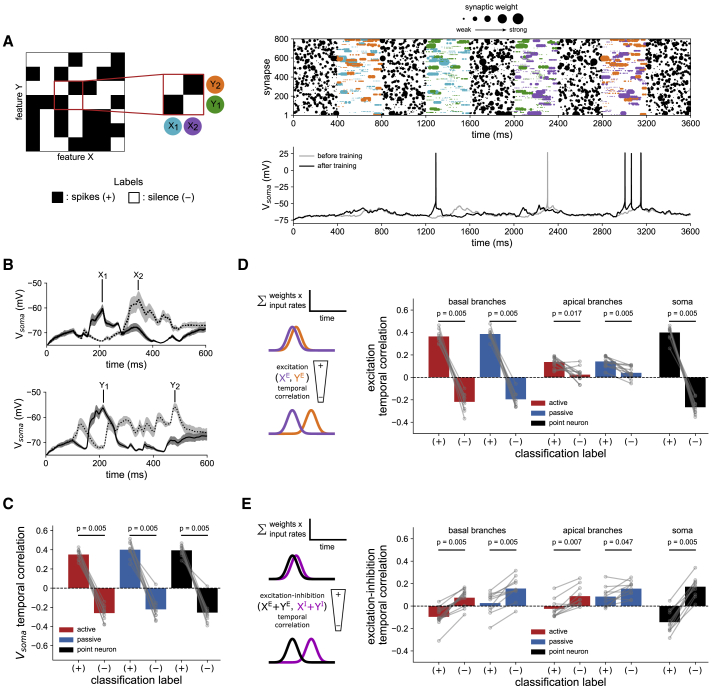
Synaptic plasticity can shape subthreshold potentials to implement a temporal feature-binding strategy (A) Example simulation of a model trained on the 7 × 7 association task in the optimal precisely timed burst input condition. The response on a nonlinear 2 × 2 subset of the task is shown, as defined by the classification labels (left). Each combination of features is presented in turn, interspersed with background noise. The markers in the raster are scaled in proportion to synaptic weight. For clarity, only input to excitatory synapses is shown. (B) Example of average subthreshold somatic membrane potentials arising from presentation of input features in isolation, with somatic spiking blocked. Shaded area is SD from 20 presentations. The components $X_{i}$ and $Y_{i}$ correspond to those simulated in (A). Note that $X_{1}$ and $Y_{1}$ will sum constructively to produce a spike at ∼200 ms, but $X_{1}$ and $Y_{2}$ will not. (C) Across all models, after training the subthreshold potentials arising from pairs forming preferred associations are temporally correlated, whereas those arising from pairs forming nonpreferred associations are anticorrelated. (D) Left: analogous to the spatial clustering strategy of Figure 4, over learning, synaptic weights evolve to temporally align patterns of excitation to bind preferred associations. $X^{\text{E}}$ and $Y^{\text{E}}$ represent excitatory input from two features. Right: correlations between temporal profiles of excitation (weighted input rates, summed over synapses) from pairs forming preferred or nonpreferred associations. (E) As in (D) but for the alignment of excitation and inhibition. $X^{\text{I}}$ and $Y^{\text{I}}$ represent inhibitory input from two features. Excitation-inhibition correlations are calculated after summing the excitatory and inhibitory contributions of each feature component in a pair. Weighted excitatory and inhibitory input is aligned on nonpreferred associations, serving to suppress somatic output. Bars denote means (averaged over 20 presentations of each association, then over label types); p values are from two-tailed Wilcoxon signed-rank tests between groups for n = 10 independent replications.

In Figure 6A, we show an example simulation of the active model in the optimal input condition solving a nonlinear subset of the task, analogous to Figure 3A. To understand the implementation, we examined the subthreshold potentials and synaptic weights of the trained models as before. Simulating the subthreshold response to individual feature components revealed a common temporal strategy. After training, the time courses of somatic responses are shaped such that only preferred pairs of features will sum constructively (Figure 6B). For all models, the somatic responses to features forming preferred associations are therefore temporally correlated, whereas responses to features forming nonpreferred associations are temporally anticorrelated (Figure 6C). Analogous to the rate-coded case (Figure 4), the implementation can be understood at the synaptic level in terms of the profiles of input that have been shaped through plasticity. For each feature component and dendritic domain we computed a temporal profile of excitation and inhibition as a time-dependent sum over synapses, scaled by synaptic weights. In the point neuron model, temporal profiles of excitation are positively correlated for preferred associations and negatively correlated for nonpreferred associations (Figure 6D). The temporal correlation between inhibition and excitation also differs with association type, but with a reversal of sign (Figure 6E). In the active and passive models, in which the influence of inhibition is spatially restricted within the tree, we find that this strategy is implemented within each dendritic domain (Figures 6D and 6E). Simulating the response to basal and apical input separately and comparing somatic membrane potentials reveals that these locally computed signals are also coordinated globally. Subthreshold potentials arising from input to each domain are preferentially aligned on preferred associations (active, 0.38 ± 0.1/0.04 ± 0.09; passive, 0.27 ± 0.12/0.21 ± 0.09; mean ± SD of temporal correlation between somatic membrane potentials from basal and apical input for preferred/nonpreferred associations), meaning that responses can be learned that are shaped locally by dendritic inhibition before appropriate summation at the soma.

In summary, these results show that from a random basis of synaptic input bursts of suitable density and precision, reliable analog signals can be constructed and selectively combined for computation. In contrast to the rate-coded scheme, this optimal representation allows binding of input features through integration in either single or multiple compartments. However, as performance was consistently high across all models in our simulations, further contributions of dendritic processing were unresolved. We now show that as this input regime contains information in both rates and spike timing, spatial processing can also be synergistically recruited as the capacity of the temporal processing strategy is reached.

### Synergistic recruitment of spatial and temporal processing

**Figure 7:**
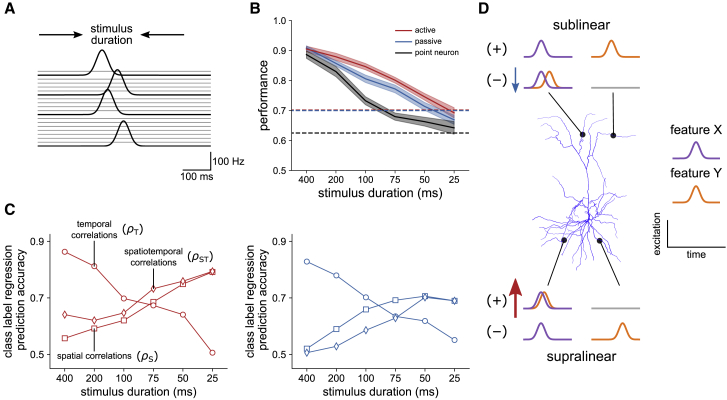
Synergistic recruitment of spatial and temporal processing (A) Schematic of presynaptic rates underlying the precisely timed burst input scheme with compressed stimulus presentation time. (B) Average model performance after training as a function of stimulus duration for ten independent replications per condition. Dashed lines are average performance under a rate code of the same sparseness and time-averaged maximum rate (20 Hz), presented for 400 ms. Note that the x axis is not a linear scale. Shaded areas are SEM. (C) Analysis of the relative contribution of spatial and temporal processing as a function of stimulus duration. The signature of each strategy is imprinted on the synaptic weights through learning, allowing the classification label of a given association pair to be predicted from knowledge of the weights and input rates. Plots show the prediction accuracy of logistic regression models fitted to predict the classification labels on the basis of correlations between spatial ($\rho_{\text{S}}$, squares), temporal ($\rho_{\text{T}}$, circles), and spatiotemporal ($\rho_{\text{ST}}$, diamonds) input profiles after training for the active (red) and passive (blue) models. Spatiotemporal correlations in the active model are more predictive of class labels than spatial correlations alone, implying a local organization of temporal signals within individual branches. (D) Schematic of spatiotemporal feature-binding strategies. Traces represent the excitation of dendritic branches with weighted input from two stimulus features. With supralinear integration, the response to preferred associations (+) can be synergistically enhanced by tuning weights such that excitation is both clustered and synchronous (denoted by red arrow). Input from nonpreferred associations (−) should instead be dispersed and asynchronous. Feature binding with sublinear integration demands dispersed, synchronous input from preferred associations and clustered, asynchronous input to suppress responses to nonpreferred associations. Although less compatible than supralinear processing, local sublinear integration could compensate on branches where temporal segregation is incomplete (blue arrow). Inhibitory input can also act in both cases to sharpen temporal responses and aid suppression of (−) pairs (not shown). See also Figure S6.

To understand what can be learned by a single neuron beyond computations involving purely spatial or temporal processing, we challenged the models with the 7 × 7 task, though under progressively shorter durations of stimulus presentation to gradually saturate the temporal capacity (Figure 7A). The performance of all models decreased with decreasing stimulus duration, reaching that of an equivalent rate code as the duration was reduced below the width of a single input burst (Figure 7B). The performance of the point neuron, however, which represents the limit of a purely temporal strategy, fell off the most rapidly. At intermediate durations, the active and passive models performed well above the lower bound represented by the equivalent rate code, even as the point neuron dipped below (active, 80% ± 3%; passive, 77% ± 4%; point neuron, 67% ± 4%; mean ± SD for 75 ms duration). This suggests a concurrence of spatial and temporal processing, enhancing performance beyond that of either strategy alone.

The analysis of trained models in Figures 4C and 4D and Figures 6D and 6E shows that distinct signatures of the spatial and temporal feature-binding strategies are imprinted on the synaptic weights through learning. We repeated this analysis to determine the contributions of spatial and temporal processing across stimulus durations. As above, we computed weighted spatial and temporal input correlations for all association pairs, separately for each dendritic domain, and for excitation and inhibition. We use the shorthand $\rho_{\text{S}}$ (spatial correlations) and $\rho_{\text{T}}$ (temporal correlations) to refer to these groups of measurements. We find that differences in $\rho_{\text{T}}$ between pairs of preferred and nonpreferred associations decrease with stimulus duration for all models (Figures S6A and S6B). In the active and passive models, this apparent reduction in temporal processing power is accompanied by a graded increase in spatial organization, revealed by differences in $\rho_{\text{S}}$, such that both signatures are present where the advantage over the point neuron is greatest (Figure S6C). We quantified this by fitting regression models to predict the classification label of each association pair from $\rho_{\text{S}}$ and $\rho_{\text{T}}$ (Figure 7C). This analysis reveals two contributions to the superior performance of the active and passive models. Sensitivity to both spatial and temporal structure gives the opportunity to use one strategy when the other would fail because of the statistics of a particular input pattern. Spatial and temporal processing can also be jointly recruited to act within a single pattern presentation.

Regression prediction accuracy is highest when regressing classification labels on $\rho_{\text{T}}$ for long durations and $\rho_{\text{S}}$ for short durations, as expected (Figure 7C). For intermediate durations, both are approximately equally effective. Pooling over the 75 ms and 100 ms conditions, we find a fraction of association labels can be correctly predicted from $\rho_{\text{T}}$, but not from $\rho_{\text{S}}$ (active, 25%; passive, 22% of labels). On this set (as defined by the active model) the average task performance of the point neuron model was 77%. A similar fraction can be correctly predicted from $\rho_{\text{S}}$, but not $\rho_{\text{T}}$ (active, 22%; passive, 23% of labels). On this set the task performance of the point neuron was reduced to 63%, close to the rate-coded lower bound (Figure 7B). These two groups reflect different subsets of associations where either the spatial or temporal strategy is implemented in isolation. However, the labels of a large fraction of associations can be correctly predicted from $\rho_{\text{T}}$ and also separately from $\rho_{\text{S}}$ (active, 44%; passive, 45% of labels), reflecting another subset in which both forms of processing are present. We explored this further by predicting labels from learned spatiotemporal input correlations, $\rho_{\text{ST}}$, computed by concatenating the temporal profiles of input to individual branches. For the active model, $\rho_{\text{ST}}$ is uniformly more predictive of class label than $\rho_{\text{S}}$ alone (Figure 7C), driven mostly by weights in basal dendrites. This implies that learning has organized temporal signals even within single dendritic branches. Clustered and synchronous excitation can thereby strongly engage supralinear integration to bind preferred associations (Figure 7D). By contrast, prediction accuracy with $\rho_{\text{ST}}$ in the passive model was degraded. Here, the spatial and temporal strategies are in opposition, as preferred associations demand dispersed yet synchronous input, whereas nonpreferred associations demand clustered yet asynchronous input (limiting engagement of dendritic nonlinearities). Although local sublinear processing could serve to compensate on nonpreferred associations where temporal segregation is incomplete (Figure 7D), the passive model lacks the inherent compatibility of the active model for spatiotemporal integration on this task, which may explain the consistent performance gap between the two (Figure 7B).

We conclude that with synaptic plasticity acting on spatiotemporal input to dendrites, complementary processing strategies can be flexibly selected, and even combined, to solve a challenging nonlinear computational task. When spatial and temporal features of synaptic input patterns both carry information about a stimulus, supralinear dendritic integration provides an ideal foundation for exploiting their interaction.

## Discussion

Nonlinear dendritic integration enables single neurons to perform sophisticated computations on their inputs. For neural circuits to capitalize on this processing capacity beyond what can be hard-wired during development requires dendritic computations to be learned. We have shown how this can be efficiently accomplished through synaptic plasticity. Through an extension of dendritic cable theory, we quantified for the first time how the spiking output of a neuron is influenced by small changes in the strengths of its many interacting synaptic inputs. We used this analysis to develop a supervised spike-timing and voltage-dependent learning rule that optimally adjusts synaptic weights to control the input-output function of a detailed pyramidal neuron model. Training the model to perform a nonlinear classification task, we demonstrated that the biophysical properties of dendrites can be harnessed without structured connectivity, which greatly expands the repertoire of dendritic computations that can be exploited by the brain. Computations that use high-order spatial or temporal features of random synaptic input patterns can be learned through the same form of plasticity, yielding synaptic weight distributions that engage supralinear or sublinear dendritic integration or precisely control the time course of subthreshold potentials. When information is encoded in both the rates and spike timing of the input, spatial and temporal processing strategies can be combined to maximize the computational power of single neurons.

The foundation of our approach is a precise mathematical description of the influence of synaptic weight changes on somatic output. Despite the inherent complexity of dendritic integration, we found this information is available to individual synapses through a simple functional dependence on synaptic activation time and dendritic voltage. A neuron can therefore optimally update its synaptic weights using local signals, while still accounting for nonlinear interactions throughout the dendritic tree. Encapsulating this insight in a supervised learning rule allowed us to explore principles of single neuron computation under a range of input conditions and model assumptions. That very different computations were learned with rate or temporal input, and even in distinct dendritic domains, demonstrates the sensitivity of our learning rule and its general applicability. The algorithmic perspective brought by our approach complements previous theoretical studies that have invoked spike-timing and voltage-dependent plasticity on the basis of experimental observations (Legenstein and Maass, 2011; Clopath et al., 2010; Bono and Clopath, 2017; Ebner et al., 2019). Although we have not sought to reproduce the results of specific experiments, our rule is biologically grounded insofar as it operates within the biophysical constraints imposed by our detailed model and weight updates are computed from variables that are plausibly accessible to dendritic synapses. We acknowledge, however, that several biological details of the implementation have been left unspecified. In particular, we have modeled the supervisory signal at an abstract level; to make the underlying assumptions explicit, a more detailed treatment in future work could include a specific circuit for neuromodulatory control (Gütig, 2014). Similarly, the implementation of the local plasticity kernels could be developed by modeling signaling cascades at the synapse that process pre- and postsynaptic spike times and the local dendritic voltage (Ebner et al., 2019). To further elucidate connections between the algorithmic and biological aspects of synaptic plasticity, another important extension of our analysis will be to characterize how the influence of synaptic weight changes depends on the wider range of dendritic mechanisms found throughout the brain. We hypothesize that, among other factors, experimentally observed differences in plasticity induction within and across cell types (Sjöström et al., 2008) may reflect specializations to allow optimal assignment of synaptic credit from local dendritic signals.

Training the model on a rate-coded feature-binding task, we showed that a single neuron can learn to implement nonlinear functions of randomly distributed input by engaging dendritic nonlinearities. Therefore, not only do pyramidal neurons share structural similarities with multilayer artificial networks (Poirazi et al., 2003b; Jadi et al., 2014), but they can capitalize on this foundation without requiring special connectivity rules or structural plasticity. Modulo the remaining mechanistic questions detailed above, this flexibility would reduce the developmental burden of precise axonal targeting and allow the output of a neuron to easily adapt to changes in input tuning or network demands. The learned implementation differs from the branch-strength potentiation modeled by Legenstein and Maass (2011), as this mechanism, observed in hippocampal neurons, is not a feature of our model. Instead, our model predicts a form of in-branch functional clustering (Kastellakis et al., 2015), similar to that imposed in the abstract Boolean model of Cazé et al. (2013), in which it was shown that distinct wiring schemes could take advantage of either supralinear or sublinear dendritic integration. Our results demonstrate that both possibilities can in fact emerge naturally from identical input connectivity and the same learning rule. As a consequence, we find opposite implementations in apical and basal dendrites, whereas the rules of multistage integration in these domains have previously been assumed to be the same (Jadi et al., 2014). Why sublinear processing in apical dendrites is optimal for the task is unclear; it may be that saturating voltages are required to overcome attenuation from the tuft (Moldwin and Segev, 2020). This intriguing result reinforces the importance of analyzing models built with realistic morphological constraints. More broadly, these observations and the comparable performance of the purely passive model underscore the potential of diverse forms of dendritic nonlinearity for computation (Poirazi and Mel, 2001; Ujfalussy et al., 2015). The power of hierarchical dendritic processing may extend far beyond the excitable dendrites of pyramidal cells to include dendrites exhibiting largely passive integration (Abrahamsson et al., 2012; Vervaeke et al., 2012), or where a mixture of both supralinear and sublinear integration is found within inhibitory interneurons (Tzilivaki et al., 2019).

Solving the same task under a range of temporal input schemes revealed an optimal regime of input comprising sparsely distributed bursts. Although it was feasible in this study to survey only a fraction of possible temporal coding schemes, our results highlight several general principles. First, as argued previously (Lisman, 1997; Krahe and Gabbiani, 2004), burst firing of presynaptic cells provides reliable units of information to postsynaptic cells. Earlier models trained on temporal pattern discrimination showed impressive performance when presented with precisely timed and reliable single spikes (Gütig and Sompolinsky, 2006, 2009; Schiess et al., 2016). We found that using a similar dense temporal scheme, although accounting for the stochasticity of spike generation, failed to improve upon the performance of a much simpler rate code (Figure 5E). However, performance improved dramatically when synapses were activated in bursts, ensuring robust signaling while retaining spike-timing information. Short-term synaptic dynamics, not considered here, could further accentuate this advantage (Lisman, 1997) and even allow multiple streams of information to be multiplexed within single cells (Körding and König, 2001; Naud and Sprekeler, 2018; Payeur et al., 2021). Second, as with connectivity, plasticity acting on globally unstructured temporal signals provides a general and flexible basis for computation. Well-studied structured alternatives include highly synchronous or sequential activation of synapses, which, through intrinsic biophysical mechanisms, can elicit differential responses to specific sets of inputs (Rall, 1964; Ariav et al., 2003; Branco et al., 2010; Branco and Häusser, 2011; Bhalla, 2017). We found, however, that with patterns defined by randomly timed events, our model could learn arbitrary responses by shaping the time course of subthreshold potentials. In this case, dendritic processing tuned by plasticity can decide the stimuli that are most relevant to a cell. Finally, our results reveal the remarkable computational potential that exists beyond a strict rate or temporal coding dichotomy. Dendrites confer exquisite sensitivity to both representations, and through spatiotemporal integration, processing of rate and temporal information can be synergistically combined.

Key predictions of our model can be tested experimentally without the need to measure synaptic weights or input rates. We propose experiments that, although challenging, could be performed in layer 2/3 pyramidal cells with currently available techniques. Performing chronic *in vivo* Ca^2+^ imaging of pyramidal cell populations while training animals to discriminate stimulus conjunctions as in Figure 3, we predict the emergence of individual neurons with nonlinear selectivity. This would confirm that such computations can be learned, even in early sensory areas, rather than being hard-wired through connectivity. In neurons exhibiting this selectivity, the signature of the optimal spatial strategy could then be identified through *in vivo* Ca^2+^ imaging of dendritic branches (Kerlin et al., 2019; Palmer et al., 2014). As schematized in Figure 7D, in basal dendrites, preferred associations are predicted to elicit a strong local Ca^2+^ signal from activating NMDA receptors in a small number of branches, whereas nonpreferred associations are predicted to elicit comparatively weaker responses in a larger number of branches (conversely for apical dendrites). The predicted signature of the temporal strategy could be identified through two-photon targeted patch-clamp recordings (Margrie et al., 2003; Kitamura et al., 2008) from the imaged population. In conjunction-selective cells, preferred associations are predicted to evoke a consistent peak in somatic voltage underlying a spiking response, whereas multiple subthreshold peaks are predicted for nonpreferred associations, reflecting the temporal alignment or misalignment of excitation arising from individual stimulus features.

Beyond the present study, the strength of our general approach is that it can be applied to investigate a wide range of possible computations, as well as other biophysical mechanisms and cell types. The governing Equations 1, 2, 3, and 4 are indeed easily extended to any number of active conductances distributed throughout a given dendritic morphology. Thus, although we have focused on voltage-dependent NMDA receptors as the major driver of supralinear synaptic integration (Branco and Häusser, 2011; Major et al., 2013), the computational roles of additional dendritic conductances can also be quantitatively assessed. We have taken a first step toward this goal, demonstrating an increase in classification performance in the presence of ${\text{I}}_{\text{h}}$ currents and dendritic Na^+^ spikes (Figure S2). However, a remaining challenge in this more excitable regime is finding an accurate local approximation of $\frac{\partial v_{\text{soma}}}{\partial w}$, as used for learning in the strictly NMDA-dependent active model (Figure 2B). This will likely require a dependence on additional local variables, such as high-pass-filtered voltage signals that can detect fast dendritic Na^+^ spikes. Further investigation of the impact of Na^+^ spikes on stimulus tuning (Smith et al., 2013; Goetz et al., 2021) and temporal processing (Ariav et al., 2003) is a priority for future work. An intriguing nonmonotonic dendritic nonlinearity was also recently found in human layer 2/3 neurons (Gidon et al., 2020). In principle, this inverted U-shaped response to input magnitude could allow an XOR to be implemented within a single compartment (Zador et al., 1991; Gidon et al., 2020), whereas implementation with the more common sigmoid nonlinearities of our model would require two (in a similar fashion to feature binding but with stronger recruitment of inhibition). Whether human neuron electrophysiology might confer additional benefits beyond this specific computation remains to be elucidated and would be of great interest to explore through task optimization of a detailed model, as we have done here. Applying our methods to study dendritic integration in neurons of cortical layer 5, the hippocampus, and cerebellum could also provide fresh insights into other active phenomena, such as associative interactions that produce apical tuft Ca^2+^ plateaus (Larkum et al., 1999; Bittner et al., 2015) and temporal regulation by ${\text{I}}_{\text{h}}$ currents (Magee, 1999; Angelo et al., 2007). Although we found that all of our major results were reproduced in a second layer 2/3 pyramidal cell model (Figure S7), it will be very revealing to explore how local rules of plasticity and computation may differ across cell types with distinct dendritic architecture and physiology. We suspect, for instance, that the segregated apical tuft of larger layer 5 neurons would preclude a direct generalization of the learning rule presented here. In this case, a two-stage rule may be more applicable, in which credit is assigned locally to synapses for producing apical Ca^2+^ plateaus, and also globally in proportion to the ability of the plateau to drive somatic output. Although many important questions about the implications of dendritic physiology for learning and computation remain open, the tools we have developed will allow these to now be systematically addressed.

## STAR★Methods

### Key resources table

**Table:** 

REAGENT or RESOURCE	SOURCE	IDENTIFIER
**Deposited data**		

Code for performing simulations	This paper	https://github.com/babicknell/Dendrites; https://doi.org/10.5281/zenodo.5524314
Reconstructed cell morphologies	Allen Institute for Brain Science, 2015	https://celltypes.brain-map.org; ID: 502359001, 521409057

**Software and algorithms**		

Python 3	Van Rossum and Drake, 2009	https://www.python.org
NEURON	Hines and Carnevale, 2006	https://www.neuron.yale.edu/neuron
Numpy	Harris et al., 2020	https://numpy.org
Numba	Lam et al., 2015	http://numba.pydata.org

### Resource availability

#### Lead contact

Further information and requests for resources should be directed to and will be fulfilled by the lead contact, Michael Häusser (m.hausser@ucl.ac.uk).

#### Materials availability

This study did not generate any new reagents.

### Method details

#### Biophysical model

##### Morphology and passive properties

We used two adult mouse V1 layer 2/3 pyramidal cell morphologies from the Allen Cell Types Database (Allen Institute for Brain Science, 2015). The morphology shown in Figure 1 (ID 502359001) was used for the majority of simulations. The second morphology (ID 521409057) was used to confirm the reproducibility of the results (Figure S7). Possible reconstruction errors in the form of pinched sections of dendrite were manually corrected by linear interpolation between adjacent segments, and the diameter profile of each branch was then smoothed with a moving average filter. Dendritic sections were subsampled such that each branch comprised at least two compartments, and the maximum compartment length was less than 10 μm (462 and 510 total dendritic compartments for the first and second morphology). For computational efficiency and to constrain the number of free parameters, we removed the detailed reconstructed axon and used a lumped axo-somatic compartment of radius 10 μm for action potential generation. The specific membrane capacitance $c_{\text{m}}=1\;\mu \text{F}\;{\text{cm}}^{-2}$, specific membrane resistance $r_{\text{m}}=10^{4}\;\text{Ω}\;{\text{cm}}^{2}$, axial resistivity $r_{\text{a}}=150\;\text{Ω}\;\text{cm}$, and leak conductance reversal potential $E_{\text{L}}=-75\;\text{mV}$ were set as in the layer 2/3 pyramidal neuron model of Branco et al. (2010), giving a membrane time constant $\tau_{\text{m}}=10\;\text{ms}$. We did not include dendritic spine compartments in the model due to the computational cost and uncertainty surrounding parameters such as spine-neck resistance. We confirmed that our main results did not differ qualitatively when modeling the influence of spines on membrane surface area using the common approach of scaling the dendritic capacitance and leak conductance by a factor of two (not shown) (Holmes and Rall, 1992; Hay et al., 2011).

##### Active conductances

For a biophysical spiking mechanism, we included fast transient ${\text{Na}}^{+}$ channels (reversal potential $E_{\text{Na}}=50\;\text{mV}$, maximum conductance $g_{\text{Na}}=80\;\text{mS}\;{\text{cm}}^{-2}$) and fast delayed-rectifier ${\text{K}}^{+}$ channels (reversal potential $E_{\text{K}}=-80\;\text{mV}$, maximum conductance $g_{\text{K}}=40\;\text{mS}\;{\text{cm}}^{-2}$) in the axo-somatic compartment. Slow persistent ${\text{K}}^{+}$ channels (reversal potential $E_{\text{K}}=-80\;\text{mV}$, maximum conductance $g_{{\text{K}}_{\text{m}}}=3\;{\text{mS}\;\text{cm}}^{-2}$, adaptation time-constant $\tau_{{\text{K}}_{\text{m}}}=200\;\text{ms}$) were included for spike-rate adaptation (Pospischil et al., 2008). Channel kinetics were implemented with standard models using the Hodgkin-Huxley formalism, as described by Pospischil et al. (2008) (see also (Equation 8), (Equation 9), (Equation 10), (Equation 11), (Equation 12)).

In the set of simulations exploring the role of intrinsic dendritic excitability (Figure S2), the ${\text{Na}}^{+}$ and ${\text{K}}^{+}$ conductances were extended throughout the dendritic tree (dendritic maximum conductances: $g_{\text{Na}}=2\;\text{mS}\;{\text{cm}}^{-2}$, $g_{\text{K}}=1\;\text{mS}\;{\text{cm}}^{-2}$, $g_{{\text{K}}_{m}}=0.15\;\text{mS}\;{\text{cm}}^{-2}$). A uniform density of HCN channels was also included in both the dendritic and axo-somatic compartments (reversal potential $E_{\text{HCN}}=-45\;\text{mV}$, maximum conductance $g_{\text{Ih}}=0.1\;\text{mS}\;{\text{cm}}^{-2}$) using the model of Kole et al. (2006). Dendritic conductance parameters were tuned such that the model produced fast dendritic ${\text{Na}}^{+}$ spikes in response to synaptic input (Smith et al., 2013), and a voltage sag of ∼1−2mV in response to hyperpolarizing current injection, consistent with deeper mouse layer 2/3 pyramidal neurons (Kalmbach et al., 2018).

##### Synaptic conductances

Excitatory synapses with AMPA and NMDA conductances, and inhibitory synapses with GABA conductances were modeled with double-exponential activation kinetics. That is, for a presynaptic spike arriving at time $t_{0}$, the time course of the activation for $t\geq t_{0}$ is given by

(Equation 6) $$g_{\text{syn}}\left(t\right)=\frac{1}{g_{\text{max}}}\left(e^{-\left(t-t_{0}\right)/\tau^{\text{d}}}-e^{-\left(t-t_{0}\right)/\tau^{\text{r}}}\right)$$

where $\tau^{\text{r}}$ and $\tau^{\text{d}}$ are rise and decay time constants specific to each conductance type, and $g_{\text{max}}$ is a normalization factor that ensures $g_{\text{syn}}$ peaks at a maximum of 1. The time-dependent activation function is scaled by a synaptic weight to determine the conductance. Weights $w_{i}$ were specific to each synapse and variable within the simulations. For excitatory synapses, the weight describes a combined NMDA and AMPA conductance, with a fixed NMDA/AMPA ratio γ=2. Rise and decay time constants for each of the conductance types were as follows. AMPA: $\tau_{\text{A}}^{\text{r}}=0.1\;\text{ms}$, $\tau_{\text{A}}^{\text{d}}=2\;\text{ms}$; NMDA: $\tau_{\text{N}}^{\text{r}}=2\;\text{ms}$, $\tau_{\text{N}}^{\text{d}}=75\;\text{ms}$; GABA: $\tau_{\text{G}}^{\text{r}}=1\;\text{ms}$, $\tau_{\text{G}}^{\text{d}}=5\;\text{ms}$, similar to previous work (Doron et al., 2017).

In the active and point neuron models, the NMDA voltage dependence was modeled by multiplying the time-dependent conductance by a local-voltage-dependent sigmoid

(Equation 7) $$\sigma_{\text{N}}\left(v\right)=\frac{1}{1+Ce^{-\rho v}},$$

as described by Jahr and Stevens (1990). We used parameters C=1/3.75 and ρ=0.062 to define the shape of the nonlinearity. While some recent studies have used steeper nonlinearities (Poleg-Polsky, 2015; Doron et al., 2017) or more complex multi-state receptor models (Branco et al., 2010), in our morphologies, we found that Equation 7 with the stated parameters was sufficient to produce the characteristic sigmoid response to synaptic input (Figure 1). The passive model was constructed by setting $\sigma_{N}\left(v\right)=1$ and leaving all other biophysical parameters unchanged. The reversal potentials for excitatory and inhibitory synapses were $E_{\text{E}}=0\;\text{mV}$ and $E_{\text{I}}=-75\;\text{mV}$, respectively.

##### Dynamics and variational equations

To simplify the notation, we provide the model equations for the general case in which ${\text{Na}}^{+}$ and ${\text{K}}^{+}$ conductances are extended throughout the entire dendritic tree. The model simulated in the main text in which these conductances are restricted to the axo-somatic compartment (summarized by Equations 1, 2, 3, and 4), is recovered by setting the dendritic conductance parameters ($g_{{\text{Na}}_{i}}$, $g_{{\text{K}}_{i}}$, $g_{{\text{Km}}_{i}}$ for i>0) to zero. For a given pattern of presynaptic input, the dynamics of the voltage in each compartment $v_{i}$ evolve by the coupled system of equations

(Equation 8) $$c_{m}\dot{v_{i}}=-\underset{j}{\sum }H_{ij}^{E}w_{j}^{\text{E}}\left(\frac{1}{1+\gamma }g_{j}^{\text{A}}\left(t\right)+\frac{\gamma }{1+\gamma }g_{j}^{\text{N}}\left(t\right)\sigma_{\text{N}}\left(v_{i}\right)\right)\left(v_{i}-E_{\text{E}}\right)-\underset{k}{\sum }H_{ik}^{I}w_{k}^{\text{I}}g_{k}^{\text{G}}\left(t\right)\left(v_{i}-E_{\text{I}}\right)-g_{{\text{Na}}_{i}}m_{i}^{3}h_{i}\left(v_{i}-E_{\text{Na}}\right)-g_{{\text{K}}_{i}}n_{i}^{4}\left(v_{i}-E_{\text{K}}\right)-g_{{\text{Km}}_{i}}p_{i}\left(v_{i}-E_{\text{K}}\right)-g_{\text{L}}\left(v_{i}-E_{\text{L}}\right)+\underset{l}{\sum }G_{il}v_{l}$$

(Equation 9) $${\dot{m}}_{i}=\alpha_{m}\left(v_{i}\right)\left(1-m_{i}\right)-\beta_{m}\left(v_{i}\right)m_{i}$$

(Equation 10) $${\dot{h}}_{i}=\alpha_{h}\left(v_{i}\right)\left(1-h_{i}\right)-\beta_{h}\left(v_{i}\right)h_{i}$$

(Equation 11) $${\dot{n}}_{i}=\alpha_{n}\left(v_{i}\right)\left(1-n_{i}\right)-\beta_{n}\left(v_{i}\right)n_{i}$$

(Equation 12) $${\dot{p}}_{i}=\alpha_{p}\left(v_{i}\right)\left(1-p_{i}\right)-\beta_{p}\left(v_{i}\right)p_{i}$$

The first two sums on the right hand side of Equation 8 describe the excitatory and inhibitory synaptic currents. The matrix with elements $H_{ij}^{E}$ projects excitatory conductances with weights $w_{j}^{E}$ into the compartments in which the synapses are located, indexed by *i*, and normalizes the resulting currents by compartment surface area to give the appropriate change in voltage. Similarly, the inhibitory currents are described by weights $w_{k}^{I}$ and projection $H_{ik}^{I}$. The functions $g_{j}^{A}\left(t\right)$, $g_{j}^{N}\left(t\right)$, and $g_{k}^{G}\left(t\right)$ denote the time-dependent activation of AMPA, NMDA and GABA conductances by input spike trains, constructed by linear summation of the responses to individual spikes described by Equation 6. The NMDA/AMPA ratio γ controls the relative contributions of excitatory conductance types. The subsequent three terms describe the active ${\text{Na}}^{+}$ and ${\text{K}}^{+}$ conductances, expressed here in generality for both the somatic and dendritic compartments. The final two terms describe leak and axial conductances, with the matrix G encoding the branching structure and geometry of the dendritic tree (see Dayan and Abbott (2001); Chapter 6). Finally, (Equation 9), (Equation 10), (Equation 11), (Equation 12) describe the kinetics of the Hodgkin-Huxley gating variables for the active conductances. Variables $m_{i}$ and $h_{i}$ control the transient ${\text{Na}}^{+}$ conductance in compartment *i*, $n_{i}$ controls the fast ${\text{K}}^{+}$ conductance, and $p_{i}$ controls the slow ${\text{K}}^{+}$ conductance. The voltage-dependent rates for each case, $\alpha_{\mu }\left(v\right)$ and $\beta_{\mu }\left(v\right)$ for μ=m,h,n,p, are as described in Pospischil et al. (2008), section 2.2.

Variational equations for the system are derived by taking partial derivatives of (Equation 8), (Equation 9), (Equation 10), (Equation 11), (Equation 12) with respect to any parameters of interest (similar to the point-neuron parameter-fitting technique of Doya et al. (1994)). When solved numerically in parallel with the model dynamics described above, these equations track how small perturbations to parameters are propagated through time and the morphology to influence the voltage throughout the tree. Here, we consider the excitatory and inhibitory weight parameters, with the aim of using the gradient of somatic voltage with respect to synaptic weights to guide learning.

We denote by $\delta v_{j}^{i}=\frac{\partial v_{i}}{\partial w_{j}}$ the partial derivative of the voltage in compartment *i* by synaptic weight $w_{j}$ (similarly for the gating variables). The component with index i=0 corresponds to the axo-somatic compartment, which we often express using the notation $\frac{\partial v_{\text{soma}}}{\partial w}$, suppressing the index for the weight when the specific identity of a synapse is not important.

For the excitatory weights,

(Equation 13) $$c_{m}\dot{\delta v_{j}^{i}}=-H_{ij}^{E}\left(\frac{1}{1+\gamma }g_{j}^{\text{A}}\left(t\right)+\frac{\gamma }{1+\gamma }g_{j}^{\text{N}}\left(t\right)\sigma_{\text{N}}\left(v_{i}\right)\right)\left(v_{i}-E_{\text{E}}\right)-[\underset{j^{\prime }}{\sum }H_{ij^{\prime }}^{E}w_{j^{\prime }}^{\text{E}}(\frac{1}{1+\gamma }g_{j^{\prime }}^{\text{A}}(t)+\frac{\gamma }{1+\gamma }g_{j^{\prime }}^{\text{N}}(t)\sigma_{\text{N}}(v_{i})+\frac{\gamma }{1+\gamma }g_{j^{\prime }}^{\text{N}}(t){\sigma_{\text{N}}}^{\text{′}}(v_{i})(v_{i}-E_{\text{E}}))+g_{{\text{Na}}_{i}}m_{i}^{3}h_{i}+g_{{\text{K}}_{i}}n_{i}^{4}+g_{{\text{Km}}_{i}}p_{i}+g_{L}]\delta v_{j}^{i}-3g_{{\text{Na}}_{i}}m_{i}^{2}h_{i}\left(v_{i}-E_{\text{Na}}\right)\delta m_{j}^{i}-g_{{\text{Na}}_{i}}m_{i}^{3}\left(v_{i}-E_{\text{Na}}\right)\delta h_{j}^{i}-4g_{{\text{K}}_{i}}n_{i}^{3}\left(v_{i}-E_{\text{K}}\right)\delta n_{j}^{i}-g_{{\text{Km}}_{i}}\left(v_{i}-E_{\text{K}}\right)\delta p_{j}^{i}+\underset{l}{\sum }G_{il}\delta v_{j}^{l}$$

(Equation 14) $$\dot{\delta m_{j}^{i}}=\left({\alpha_{m}}^{\text{′}}\left(v_{i}\right)\left(1-m_{i}\right)-{\beta_{m}}^{\text{′}}\left(v_{i}\right)m_{i}\right)\delta v_{j}^{i}-\left(\alpha_{m}\left(v_{i}\right)+\beta_{m}\left(v_{i}\right)\right)\delta m_{j}^{i}$$

(Equation 15) $$\dot{\delta h_{j}^{i}}=\left({\alpha_{h}}^{\text{′}}\left(v_{i}\right)\left(1-h_{i}\right)-{\beta_{h}}^{\text{′}}\left(v_{i}\right)h_{i}\right)\delta v_{j}^{i}-\left(\alpha_{h}\left(v_{i}\right)+\beta_{h}\left(v_{i}\right)\right)\delta h_{j}^{i}$$

(Equation 16) $$\dot{\delta n_{j}^{i}}=\left({\alpha_{n}}^{\text{′}}\left(v_{i}\right)\left(1-n_{i}\right)-{\beta_{n}}^{\text{′}}\left(v_{i}\right)n_{i}\right)\delta v_{j}^{i}-\left(\alpha_{n}\left(v_{i}\right)+\beta_{n}\left(v_{i}\right)\right)\delta n_{j}^{i}$$

(Equation 17) $$\dot{\delta p_{j}^{i}}=\left({\alpha_{p}}^{\text{′}}\left(v_{i}\right)\left(1-p_{i}\right)-{\beta_{p}}^{\text{′}}\left(v_{i}\right)p_{i}\right)\delta v_{j}^{i}-\left(\alpha_{p}\left(v_{i}\right)+\beta_{p}\left(v_{i}\right)\right)\delta p_{j}^{i},$$

where ${\sigma_{\text{N}}}^{\text{′}}\left(v\right)$, ${\alpha_{\mu }}^{\text{′}}\left(v\right)$ and ${\beta_{\mu }}^{\text{′}}\left(v\right)$ denote derivatives with respect to voltage. The expression for inhibitory weights is similar.

In a subset of the simulations presented in Figure S2 we also include voltage-dependent HCN channels in the axo-somatic and dendritic compartments. For this we used the Hodgkin-Huxley type channel model of Kole et al. (2006), which describes the channel kinetics in terms of a single gating variable that we denote by *q*. The HCN conductance can be incorporated into the model in the same manner as the other active conductances, by adding the additional term $-g_{{\text{Ih}}_{i}}q_{i}\left(v_{i}-E_{\text{hcn}}\right)$ to Equation 8 and an additional first order equation for the gating variable analogous to (Equation 9), (Equation 10), (Equation 11), (Equation 12). The associated terms that appear in the variational equations are computed by taking partial derivatives. Note that for channel models constructed using the Hodgkin-Huxley formalism these operations are formulaic, allowing our general approach to be extended to additional conductance mechanisms.

We solve the system numerically using a custom implicit Euler solver written in Python 3 (Van Rossum and Drake, 2009), using Numpy (Harris et al., 2020) and Numba (Lam et al., 2015). Theoretical background for the implementation was drawn from Hines (1984); Dayan and Abbott (2001); Hines and Carnevale (2006), and the accuracy of solutions to (Equation 8), (Equation 9), (Equation 10), (Equation 11), (Equation 12) was validated during development using NEURON 7.5 (Hines and Carnevale, 2006). After using this custom simulator to derive the local plasticity kernels as described below, simulations of the model were performed using NEURON 7.5 for computational efficiency.

#### Synaptic input patterns

To explore the space of possible input regimes, we parameterized a model for generating random input patterns for the feature-binding task. A total number of $N_{\text{syn}}$ synapses were randomly divided into two equally sized subsets, representing presynaptic populations associated with two feature classes (described as feature *X* and feature *Y*). Input patterns for specific features within a class $\left(X_{1},X_{2},\ldots \right)$ were defined as vectors of time dependent rates for the $N_{\text{syn}}/2$ synapses assigned to the class. Input patterns were realized in simulations as non-homogeneous Poisson spike trains, generated by rejection sampling. Unless stated otherwise, each input pattern consisted of 100 ms of background activity at constant rate $\lambda_{0}=1.25\;\text{Hz}$ for all synapses, followed by a T=400ms stimulus presentation between times $t_{\text{on}}$ and $t_{\text{off}}$. To constrain the parameter space, the time- and population-averaged firing rate of the stimulus component was fixed at $\lambda_{\text{pop}}=2.5\;\text{Hz}$ per synapse to give an average of one presynaptic spike per synapse over the 400 ms stimulus presentation.

Two parameters define the characteristics of a pattern: the time-averaged firing rate of active synapses $\lambda_{\text{syn}}$, and the number of precisely timed events *K*. To satisfy the imposed constraint that ensemble-averaged activity is maintained at a constant level, $\lambda_{\text{syn}}$ also determines the sparseness of activity – few synapses active with high firing rates, or many active with low firing rates. Synapses were thus assigned time-averaged rates $\bar{\lambda }=\lambda_{\text{syn}}$ with probability $\lambda_{\text{pop}}/\lambda_{\text{syn}}$, or $\bar{\lambda }=0$ otherwise. Precisely timed events were implemented as Gaussian bumps of elevated firing rate centered at uniformly distributed times. To ensure physiological instantaneous firing rates, Gaussian widths were scaled with the number of presynaptic spikes expected to occur during an event (which depends on $\bar{\lambda }$) from an initial value of $\sigma_{0}=2.5\;\text{ms}$. Altogether, the stimulus-dependent component of the rate function for the *i*th synapse, $\lambda_{i}\left(t\right)$, was initialized as

(Equation 18) $$\lambda_{i}\left(t\right)=\left\{ \begin{array}{l} {\bar{\lambda }}_{i},\;K=0\\ \frac{\bar{\lambda_{i}}T}{K}\overset{K}{\underset{k=1}{\sum }}g\left(t-t_{i}^{k},\sigma_{0}\frac{\bar{\lambda_{i}}T}{K}\right),\;K>0 \end{array}\right.$$

(Equation 19) $$\frac{{\bar{\lambda }}_{i}}{\lambda_{\text{syn}}}∼\text{Bernoulli}\left(\frac{\lambda_{\text{pop}}}{\lambda_{\text{syn}}}\right)$$

(Equation 20) $$t_{i}^{k}∼\text{unif}\left(t_{\text{on}},t_{\text{off}}\right).$$

The multiplicative factor $\bar{\lambda_{i}}T/K$ in the K>0 case in Equation 18 scales the time-dependent rates such that the integral over $\left[t_{\text{on}},t_{\text{off}}\right]$, ignoring small boundary effects, is equivalent to that when K=0. When pairs of features are presented together, for instance $X_{1}$ and $Y_{1}$, the expected number of presynaptic spikes over all synapses is thus $N_{\text{syn}}\lambda_{\text{pop}}T$ for all choices of parameters.

#### Plasticity kernels

We constructed plasticity kernels to be used in the learning algorithm (Figure 2B; Figures S1A–S1D) by approximating the output of (Equation 13), (Equation 14), (Equation 15), (Equation 16), (Equation 17) in terms of the local dendritic voltage and synaptic activation time. Specifically, we approximated the influence of synaptic weight changes on the somatic membrane potential at times immediately preceding somatic spikes, $\frac{\partial v_{\text{soma}}}{\partial w}\left(t_{\text{spike}}\right)$. In this way, when the kernels are used to update synaptic weights at the time of somatic spikes, synapses are selectively modified relative to their ability to enhance or suppress somatic output in response to a given input pattern.

To construct the approximation, for each of the active, passive and point neuron models we ran 5000 10 s simulations using randomly generated Poisson input. In each simulation, input rates were drawn independently for each synapse from a lognormal distribution $\lambda_{i}∼\text{lognormal}\left(0,1\right)$, yielding a bombardment of synaptic input featuring both isolated single spikes and high frequency spike trains. Synaptic weights were set for excitatory and inhibitory synapses by randomly perturbing the initial values $w^{\text{E}}=0.6\;\text{nS}$ and $w^{\text{I}}=0.8\;\text{nS}$, giving a range of weights ∼0.4−1nS. For the passive model, the excitatory weights were reduced by a factor of five to give a similar postsynaptic firing rate.

Guided by preliminary simulations, we focused our analysis on windows of 150 ms preceding somatic spikes. We excluded windows in which additional output spikes had occurred, to ensure we collected independent measurements (leaving $∼10^{5}$ somatic spikes for analysis in each condition). For each somatic spike we computed the influence of synaptic weight changes on the somatic voltage at the upswing of the action potential (defining $t_{\text{spike}}$ as 2 ms before the voltage crossed 0 mV). We used this time point as a functional definition of spike threshold crossing; once the autonomous voltage-dependent spiking mechanism is engaged during the upswing, the somatic voltage becomes far less susceptible to synaptic control (i.e., ‘the horse has bolted’). Integrating (Equation 13), (Equation 14), (Equation 15), (Equation 16), (Equation 17) for a detailed model is computationally expensive, so was performed over the selected 150 ms windows, rather than the entire 10 s simulation run. This was accomplished by simulating the model again over the selected window using initial conditions and input set from the same time point in the original run. We also made two small modifications in these sub-simulations, which were found during development to enable more reliable predictions about the influence of weight changes on spiking output. First, we made dummy copies of any synapses that were activated more than once, such that every synapse received a single presynaptic spike, while the input to the neuron remained functionally identical. This allowed us to compute and then approximate $\frac{\partial v_{\text{soma}}}{\partial w}$ for each individual input, rather than the value aggregated over a sequence of randomly timed inputs. Second, we blocked the action potential at the end of the sub-simulation window by setting the fast axo-somatic ${\text{Na}}^{+}$ and ${\text{K}}^{+}$ conductances to zero. This avoids occasional spuriously high $\frac{\partial v_{\text{soma}}}{\partial w}$ values arising from the steep nonlinearity of the action potential itself, but has negligible influence on the preceding sub-threshold integration that is the underlying cause. Along with the computed values of $\frac{\partial v_{\text{soma}}}{\partial w}$ for each somatic spike, we recorded the associated presynaptic spike times, synaptic weights and dendritic voltages.

For the active and passive models, we pooled the simulated data across synapse locations in basal and apical dendrites. We computed averages of $\frac{\partial v_{\text{soma}}}{\partial w}$ as a function of the difference in timing between pre- and post-synaptic spikes, and the local dendritic voltage at the time of the post-synaptic spike. In Figure 2D we show that the local voltage at the time of the post-synaptic spike encodes the level of activity of neighboring excitatory and inhibitory synapses over the past 100 ms. This is what allows our rule to selectively enhance or suppress coactive groups of synapses within a branch. Note that if plasticity were instead to depend on the voltage at the time of a presynaptic spike, much of this crucial dependence would be lost because the rule would be blind to any subsequent activity in the branch. Averages were computed after discretizing time and voltage in 1 ms and 1 mV bins. The plasticity kernels were then constructed by fitting the averaged two-dimensional functions with polynomials of degree 8 in both variables. Because the simulated data did not cover all points of the parameter space, the boundaries at v=0mV, v=−80mV and t=−1ms were first padded with zeros to allow a smooth interpolation over the whole domain. We also restricted the temporal dimension to a limit of 100 ms time difference between pre- and post-synaptic spikes, beyond which the values of the spike-triggered averages are effectively zero. After fitting, the kernels were thresholded to remove negative values from the excitatory kernels and positive values from the inhibitory kernels, which was found to improve convergence in pilot simulations. For the point neuron model, and simulations using temporal kernels (Figure 3C), we fitted the temporal spike-triggered average (Figure S1A–S1C) with a degree 10 polynomial after padding the boundary at t=−1ms. The accuracy of the fitted kernel approximations was assessed by fitting on 75% of the data and testing on the remaining 25% (Figure 2C), and was used to inform the choices of polynomial degrees used in the fits.

#### Learning rule

Our aim was to develop a supervised spike-based plasticity rule that could take advantage of the complex morphology and biophysics of pyramidal cell dendritic trees. Importantly, we sought a formulation that could be applied under diverse forms of noisy synaptic input and that did not presuppose the implementation of a given computation (for instance, by including an explicit mechanism to encourage synaptic clustering). Inspired by the tempotron (Gütig and Sompolinsky, 2006), we used a greedy algorithm that updates synaptic weights in proportion to their influence on the somatic membrane potential, $\frac{\partial v_{\text{soma}}}{\partial w}$. Although the tempotron rule is based on a single-compartment leaky integrate-and-fire neuron model, we found that the underlying principles provide a powerful heuristic for implementing spike-based learning in a detailed biophysical model. We first outline the computational task and tempotron rule, and then detail the modifications that lead to the rule represented by Equation 5 of the main text.

We consider a general binary classification problem, in which a neuron must produce at least one postsynaptic spike in response to preferred patterns of input, and remain silent for nonpreferred patterns. For the specific feature-binding task that we study, input patterns are defined by different pairs of stimulus associations. The preferred and nonpreferred classes of input patterns are denoted by the symbols (+) and (−). Upon presentation of an input pattern *p*, realized as stochastic presynaptic spiking activity, a binary variable $z_{p}$ records the output of the neuron, taking the value $z_{p}=1$ if at least one spike is emitted, and $z_{p}=0$ if the neuron remains silent. The target output is given by a label $z_{p}^{\text{∗}}$, taking the value $z_{p}^{\text{∗}}=1$ for (+) patterns and $z_{p}^{\text{∗}}=0$ for (−) patterns. We define a signed classification error as

(Equation 21) $$E_{p}=z_{p}-z_{p}^{\text{∗}},$$

which takes the value $E_{p}=0$ for correct trials, $E_{p}=-1$ for (+) pattern errors, and $E_{p}=1$ for (−) pattern errors. The goal of learning is to minimize the expected absolute value of $E_{p}$ for all input patterns. As a proxy for the expected value, we use an average over a sample of trials, defining the total classification error for the whole set of $N_{p}$ patterns by

(Equation 22) $$\bar{E}=\frac{1}{N_{p}}\underset{p}{\sum }\;\left|\bar{E_{p}}\right|,\;\bar{E_{p}}=\bar{z_{p}}-z_{p}^{\text{∗}},$$

where bars denote an average over $N_{\text{avg}}$ pattern presentations. Unless stated otherwise, we set $N_{\text{avg}}=10$.

##### Tempotron learning

For binary classification with tempotron learning, input patterns are presented sequentially over many trials and synaptic weights are modified when the neuron makes an error. Plasticity during presentation of a pattern *p* is guided by gradient descent on a single-pattern loss function that measures how far the somatic voltage deviates from spiking threshold (assumed to be fixed). In our notation, the loss function is given by

(Equation 23) $$E_{p}=\left(V_{\text{soma}}\left(t_{\text{max}}\right)-V_{\text{th}}\right)E_{p}.$$

In Equation 23, $V_{\text{th}}$ denotes the spiking threshold and $V_{\text{soma}}\left(t_{\text{max}}\right)$ denotes the voltage at the time of the first post-synaptic spike, or the maximum subthreshold voltage attained if no spikes were emitted. By definition of $E_{p}$, and assuming a spike is fired when $V_{\text{soma}}$ crosses $V_{\text{th}}$, $E_{p}=0$ on correct trials, and $E_{p}>0$ for both (+) and (−) pattern error trials.

After presentation of an input pattern, synaptic weights are updated in proportion to the negative gradient of the loss function with respect to the weights,

(Equation 24) $$\text{Δ}w_{i}\propto -\frac{\partial E_{p}}{\partial w_{i}}\approx -E_{p}\frac{\partial V_{\text{soma}}}{\partial w_{i}}\left(t_{\text{max}}\right).$$

We write Equation 24 as an approximation because it ignores the possibly discontinuous dependence of the value of $t_{\text{max}}$ itself on the weights (see Urbanczik and Senn (2009) for a detailed discussion). The first factor on the right-hand side of Equation 24 acts as a corrective supervisory signal – plasticity is gated on only on error trials, with the sign determined by the class of pattern (positive for (+) pattern errors and negative for (−) pattern errors). The second factor implements synaptic credit assignment by scaling the magnitude of weight update for each synapse by its contribution to the voltage at time $t_{\text{max}}$. Intuitively, iterating over repeated presentations of input patterns, locally optimal weight updates are applied to push peaks in the somatic voltage below threshold on (−) patterns, and to push peaks above threshold on (+) patterns.

##### Adaptive learning with dendritic synapses

The tempotron rule can be directly generalized to our biophysical model by using values of $\frac{\partial V_{\text{soma}}}{\partial w}$ computed from Equation 13. However, with dendritic synapses, this rule becomes implausibly nonlocal. In (+) pattern error trials, where no spike was emitted, $t_{\text{max}}$ must somehow be computed and transmitted to a synapse from the subthreshold activity at the soma. Furthermore, with an extended morphology, the value of $\frac{\partial V_{\text{soma}}}{\partial w}$ for a given synapse formally depends on activity in even distant dendrites. To address these issues and better satisfy the requirements of our noisy task, we make three modifications to the tempotron rule. First, we introduce an explicit mechanism that allows the timing of somatic events to be communicated to dendritic synapses. Second, we use an approximation that allows accurate synaptic credit assignment using local dendritic signals. Third, in pursuit of minimizing $\bar{E}$ (Equation 22), we replace the gating factor $E_{p}$ with modulation by the running average error $\bar{E_{p}}$. The first and third modifications are achieved using an adaptive supervisory signal that guides the learning process. Subsequently, we show that these dynamics permit a straightforward extension to an online implementation, where learning is controlled by feedback from the spiking output of the neuron.

In our approach, we assume that an external supervisory system maintains a running average of past errors, $\bar{E_{p}}$. The supervisory system guides learning by encouraging spiking when it is desired, and modulating the sign and magnitude of plasticity. On presentation of (+) patterns during training, a depolarizing teaching current is activated at the soma

(Equation 25) $$I_{\text{teach}}=\beta \left|\bar{E_{p}}\right|.$$

The teaching current could be provided through regulation of synaptic input or active conductances. In simulations, for simplicity, we use a direct somatic current injection. We set the parameter β=0.1nA, yielding a maximum current that is too small to elicit spiking on its own, but sufficient in the presence of synaptic input. When the neuron has been performing poorly, the teaching current adds a constant positive offset to the somatic potential, $V_{\text{teach}}\approx I_{\text{teach}}R_{\text{in}}$, where $R_{\text{in}}$ denotes the somatic input resistance measured in the absence of synaptic activity. This has the effect of raising otherwise-subthreshold peaks in the somatic voltage above the spiking threshold. The time of a peak, now marked by a backpropagating action potential (Stuart and Sakmann, 1994; Stuart et al., 1997), can thus be broadcast to the dendrites, and does not require secondary computation or transmission after the pattern has been presented. As performance improves, the teaching current gradually decreases until positive classifications are made without assistance.

During presentation of either (+) of (−) patterns, synaptic weights are modified whenever a somatic action potential is fired. The specific contribution of each synapse to the somatic voltage is computed by approximating the output of Equation 13 with a local plasticity kernel (Figure 2B), summed over presynaptic spikes,

(Equation 26) $$\frac{\partial V_{\text{soma}}}{\partial w_{i}}\left(t_{\text{spike}}\right)\approx \underset{k}{\sum }K_{i}\left(\text{Δ}t_{i}^{k},v_{{\text{dend}}_{i}}\right).$$

In Equation 26, the index *k* runs over presynaptic spikes that arrived at synapse *i* in the past 100 ms, $K_{i}$ is the appropriate plasticity kernel for the synapse (basal, apical; excitatory, inhibitory), $\text{Δ}t_{i}^{k}$ is the time of the *k*th synaptic input relative to the somatic spike, and $v_{{\text{dend}}_{i}}$ is the local dendritic voltage at the time of the somatic spike. Intuitively, to implement Equation 26, each synapse requires a plasticity kernel that is common among all synapses in basal and apical domains, to know that a postsynaptic spike has occurred, to know the local voltage, and to know how far in the past presynaptic spikes arrived.

Analogous to Equation 24, weight updates are then determined by the product of the locally approximated gradient of the voltage and a global scalar error term, scaled by the learning rate α (Equation 5, in the main text),

(Equation 27) $$\text{Δ}w_{i}=-\alpha \bar{E_{p}}\underset{k}{\sum }K_{i}\left(\text{Δ}t_{i}^{k},v_{{\text{dend}}_{i}}\right).$$

Using the average ${\bar{E}}_{p}$ in Equation 27 introduces adaptive dynamics to the learning process. When past performance has been consistently poor, updates are large and favor exploration of the weight space. As performance improves, the magnitudes of updates decrease, which favors refinement of the solution. Learning ceases upon minimization of $\bar{E}$. For comparison, we also tested an alternative rule in which plasticity was instead gated only by the error on the current pattern (for this purpose, positive classifications were considered as errors if assisted by the teaching current). We found that while both rate and temporally coded tasks could still be learned, performance was inconsistent and highly sensitive to the learning rate (Figure S5).

##### Training and testing procedure

Unless otherwise stated, all models were trained using the same procedure and learning rule. $N_{p}$ input patterns, divided into (+) and (−) classes, were presented in random order in each of 1000 total epochs of training (500 epochs for the parameter sweep in Figure 5C). For computational efficiency, the simulations were interrupted at the time of the first somatic spike during a pattern presentation, weights were updated using Equation 27, and the next pattern was presented. Training ended when the maximum number of epochs was reached, or after ten successive epochs without errors $\left(\bar{E}=0\right)$. With synaptic weights defined in the simulations in units of μS, we used an initial learning rate $\alpha =2\times 10^{-6}/\lambda_{\text{syn}}$, which decayed as a function of training epoch as $1/\left(1+\frac{1}{125}x\right)$. The learning rate parameters were selected based on pilot simulations using the 2×2 association task. In case of runaway growth of the weights, we enforced a maximum weight by clipping at 0.01μS, though in practice this maximum was never reached. Model training required ∼1h of CPU time on a single core for learning the 2×2 task, and ∼12h for the 7×7 task.

After training, all patterns were presented 20 times with both plasticity and the depolarizing teaching current turned off. Testing performance was quantified from these simulations as $P_{\text{test}}=1-\bar{E}$, using $N_{\text{avg}}=20$.

##### Online implementation

The history dependence of our learning rule provides a basis for an online implementation, in which errors are integrated over time within a single pattern presentation. In this case we assume that the supervisor provides only a binary classification label when a pattern is presented. All other quantities are computed online from spiking output and fed back to the neuron to guide plasticity.

The input patterns to be classified were defined as above by time series of presynaptic rates. During training, to allow for temporal integration and feedback, patterns were presented for a duration $T_{\text{train}}$, ranging from 1−4s. For the precisely timed burst input condition, this was achieved by periodic extension of the 400ms input patterns over the training presentation time (Figure S5C). All patterns were superimposed on a constant background rate of $\lambda_{0}=1.25\;\text{Hz}$ to every synapse, which remained active throughout the whole presentation period (equivalent to a background noise level of 0.5, in Figure S3B).

We augmented the model with two first-order equations that compute the classification error for the current input pattern as a function of recent spiking activity and the label $z_{p}^{\text{∗}}$,

(Equation 28) $$\tau_{r}\dot{r}=\delta_{\text{spike}}\left(t\right)-r$$

(Equation 29) $$\tau_{E}\dot{E_{p}}=\text{Θ}\left[r-r_{\text{L}}\left(1-z_{p}^{\text{∗}}\right)-r_{\text{U}}z_{p}^{\text{∗}}\right]-z_{P}^{\text{∗}}-E_{p}$$

Equation 28 computes an exponentially weighted average of the spike count over an averaging time $\tau_{r}$. The first term $\delta_{\text{spike}}\left(t\right)$ denotes an impulse applied whenever the somatic voltage cross 0 mV from below. In practice, when the system is discretized for simulations, the variable *r* is instantaneously incremented by $1/\tau_{\text{r}}$ in any time step in which a spike occurs. Equation 29 uses the sampled rate to compute an exponentially weighted average of the classification error over averaging time $\tau_{E}$. In the first term of Equation 29, Θ denotes the Heaviside step function (Θ[x]=1 for x≥0 and Θ[x]=0 for x<0), which thresholds the rate *r*, analogous to the variable $z_{p}$ in Equation 21. To enforce the spikes/silence binary classification we use two separate thresholds. For (+) patterns, when $z_{p}^{\text{∗}}=1$, the rate is compared to an upper threshold $r_{\text{U}}$. For (−) patterns, when $z_{p}^{\text{∗}}=0$, the rate is compared to a lower threshold $r_{\text{L}}$. The time-averaged error defined by Equation 29 converges to zero when the rate is stably maintained above $r_{\text{U}}$ on (+) patterns, and below $r_{\text{L}}$ on (−) patterns. In the simulations presented in Figure S5, we used parameters $\tau_{\text{r}}=1000\;\text{ms}$, $\tau_{\text{E}}=500\;\text{ms}$, $r_{\text{L}}=0.1\;\text{Hz}$, and $r_{\text{U}}=5\;\text{Hz}$.

For each presentation of an input pattern, variables *r* and $E_{p}$ are initialized at 0, and numerically integrated in parallel with the model voltage dynamics. The variable $E_{p}$ – now recording a temporal rather than trial average – determines the magnitude of the teaching current activated during (+) patterns as in Equation 25. For the first $T_{\text{train}}/2$ ms, the model is simulated without plasticity to allow the system to come to a steady state. At time $T_{\text{train}}/2$, plasticity is turned on and weight updates are applied using Equation 27 (substituting the time-averaged $E_{p}$) at the time of every somatic spike. The time at which plasticity is turned on is largely arbitrary; generally, we found that learning was more stable when adding a delay that avoids the transient dynamics that arise from initializing Equations 28 and 29 at 0, and the model at rest, for each new pattern presentation. In Figure S5D we show examples of the online learning dynamics during single pattern presentations.

Models were trained using this procedure for 500 epochs. After training, using identical conditions to the offline learning simulations, models were tested on pattern presentations of duration of $T_{\text{test}}=500\;\text{ms}$, comprising 100 ms of background input, followed by 400 ms of background and stimulus-dependent input.

#### Spatial and temporal processing analysis

##### Subthreshold integration

After training, models were simulated with the fast axo-somatic Na^+^ and K^+^ conductance parameters set to zero to block somatic spiking ($g_{{\text{Na}}_{0}}$ and $g_{{\text{K}}_{0}}$ in Equation 8).

To quantify the supralinearity and sublinearity or responses in Figures 3D, S2C, and S7G, input patterns representing individual feature components were presented individually and then together in association pairs. This was performed separately for basal and apical dendrites by setting synaptic weights to zero in one or the other domain. Somatic voltage traces were recorded and averaged over 20 replications. The nonlinearity of summation for each association was defined as the peak of the average membrane potential when both features were presented together divided by the peak of the sum of the traces from features presented separately. Peaks were computed from the second half of the 400 ms stimulus presentation time to capture the steady-state response.

To quantify the temporal alignment of responses in Figures 6C and S7H, somatic voltage traces were recorded from presentation of individual feature components and averaged over 20 replications (including both basal and apical input together for the active and passive models). The Pearson correlation was computed between average traces from each feature in an association pair, using the entire stimulus presentation time. To compare the output of the basal and apical domains for each association, pairs of features were presented together with input restricted to one or the other domain, and the Pearson correlation was computed between the average basal-input and apical-input traces.

##### Weighted input profiles

To understand the computational strategies learned by the models, we examined how the synaptic weights determine the spatial and temporal distribution of input strength. We defined a spatial profile of input by a vector with each element $S_{b}$ computed as the sum of time-averaged excitatory or inhibitory input rates to a single dendritic branch, scaled by synaptic weights $S_{b}=\underset{i}{\sum }w_{i}^{b}{\bar{\lambda }}_{i}^{b}$. Here, $w_{i}^{b}$ and ${\bar{\lambda }}_{i}^{b}$ denote the weights and rates associated with branch *b*. Similarly, replacing branches with 1 ms time steps, we defined a temporal profile of input by a time-dependent sum of rates over all synapses in a given domain (basal, apical or soma), $T\left(t\right)=\underset{i}{\sum }w_{i}\lambda_{i}\left(t\right)$. Spatiotemporal profiles were constructed by computing a temporal profile of input into each branch, $T_{b}\left(t\right)=\underset{i}{\sum }w_{i}^{b}\lambda_{i}^{b}\left(t\right)$, and then concatenating the branch time series. We use these definitions to quantify the manner in which learning has tuned the interactions between pairs of features forming associations. In Figure 4C, we quantify the spatial clustering of weighted excitatory input patterns by computing the Pearson correlation between their respective spatial input profiles. In Figure 4D, we quantify the spatial clustering of weighted excitatory and inhibitory input patterns. In this case, we first sum the excitatory profiles of each pair, separately sum the inhibitory profiles, and then compute the Pearson correlation between the total excitatory and inhibitory profiles. Similarly, in Figures 6D and 6E, we quantify the temporal alignment of weighted input patterns by computing the Pearson correlation between their respective temporal input profiles.

##### Regression of classification labels

In Figure 7 we simulate a regime of input in which both spatial and temporal processing strategies can be learned. To unpack the relative contributions, we use regression models to predict the classification label of a given association pair from the profiles of input that have been shaped by plasticity. Distinct signatures of spatial and temporal processing are shown in Figures 4 and 6. For the spatial strategy, preferred patterns are associated with positive correlations between excitatory spatial profiles (spatial clustering) and nonpreferred patterns with negative correlations (spatial dispersion). Analogously, for the temporal strategy, preferred patterns are associated with positive correlations between excitatory temporal profiles (temporal alignment) and nonpreferred patterns with negative correlations (temporal misalignment). The reverse contingencies tend to hold when comparing excitation and inhibition. In Figure 7, pooling across all simulated data for each stimulus duration, we fit separate logistic regression models to predict association label, (+) or (−), from correlations between the spatial, temporal and full spatiotemporal profiles of association pairs (using *LogisticRegression* from the Scikit-learn library in Python). Prediction accuracy was determined using leave-one-out cross validation. Regressors included correlations between profiles of excitation, and between profiles of excitation and inhibition, computed separately for basal and apical domains. In the temporal model, we also included correlations between global temporal profiles, computed as a sum over all synapses.

#### Noise robustness

Models were trained and tested under a variety of conditions to assess the robustness to multiple sources of noise: trial-by-trial spike count variability (Figure S3A), background synaptic activity (Figures S3B and S4B), mislabelling during training (Figure S3C), perturbations to synaptic weights (Figure S4C), and trial-by-trial burst timing variability (Figure S4D).

Robustness to spike count variability was tested by varying the rates of presynaptic input between 2.5−40Hz via parameter $\lambda_{\text{syn}}$ in Equation 19. Although our model for generating input patterns keeps the total population rate fixed for different values $\lambda_{\text{syn}}$ (by adapting the sparseness of activation), smaller $\lambda_{\text{syn}}$ increases the standard deviation of the number of spikes arriving at each synapse, relative to the mean. To test the dependence on spike-count variability at the presynaptic population level, for each value of $\lambda_{\text{syn}}$, we also varied the total number of synapses from $N_{\text{syn}}=500-4000$. To maintain similar output responses to the main model $\left(N_{\text{syn}}=1000\right)$ at the onset of training, initial values of synaptic weights were scaled by a multiplicative factor of $1000/N_{\text{syn}}$.

Robustness to background synaptic activity was tested by varying the background rate parameter $\lambda_{0}$. In the simulations presented in Figures 3, 4, 5, 6, and 7, $\lambda_{0}$ was set to 1.25Hz for all synapses for the first 100 ms of a simulation, and set to zero during the stimulus presentation. For the simulations in Figures S3B and S4B, $\lambda_{0}$ was varied between 0.625−5Hz, and remained at that level during the stimulus presentation. We quantify the background noise level by the ratio of the background and stimulus-dependent population rates, $\lambda_{0}/\lambda_{\text{pop}}$ (see Equations 19 and 20). When the noise level is 1, during the stimulus presentation, as many background spikes arrive across the population of $N_{\text{syn}}$ synapses as do ‘signal’ spikes that depend on the specific input pattern being presented. To maintain similar output responses at the onset of training, initial values of synaptic weights we scaled by a multiplicative factor of $\lambda_{\text{pop}}/\left(\lambda_{0}+\lambda_{\text{pop}}\right)$ to account for the additional input spikes. For the 2×2 association task (Figure S3B), these simulations were repeated with $N_{\text{syn}}=500-4000$, and initial weight values scaled by an additional factor of $1000/N_{\text{syn}}$.

Robustness to label noise was tested by varying the probability that models were given an incorrect classification label during training. For each presentation of an input pattern, the label was switched with probability $p_{\text{mislabel}}$ ranging between 0−0.2. During training, the label was used as in the noise-free case to determine both the sign of plasticity and the presence of a depolarizing current at the soma. The error term $\bar{E_{p}}$, which scales the magnitude of plasticity, was computed with reference to the noisy labels.

Robustness to synaptic weight jitter was tested by perturbing the weights of trained models with random multiplicative noise. Models were tested on 20 presentations of each input pattern used in training. Each time a pattern was presented, a noise term was drawn independently for every synapse from a uniform distribution over the interval $\left[-s_{\text{jitter}},s_{\text{jitter}}\right]$, with the scaling factor $s_{\text{jitter}}$ ranging from 0.5−2. Weights were multiplied by $\left(1+s_{\text{jitter}}\right)$, and thresholded at zero to avoid negative weights. In Figure S4C, model performance is quantified as a function of the standard deviation of the scaling factor; $s_{\text{jitter}}=1$ corresponds to a weight jitter SD of 50%.

Robustness to timing variability was tested for the precisely timed burst input condition by perturbing the times of input bursts. Models were tested on 20 presentations of each input pattern used in training. We considered two types of perturbation – shared and independent. For the shared case, each time a pattern was presented, a single noise term was drawn from a uniform distribution over the interval $\left[-t_{\text{shift}},t_{\text{shift}}\right]$ and all burst times were periodically shifted within the 400 ms stimulus presentation window by this value. In the independent case, noise terms were drawn for every synapse, and burst times were shifted independently. The parameter $t_{\text{shift}}$ was varied between 50−400ms, and the noise level quantified by the standard deviation of the noise term. Note that for a timing jitter standard deviation of 100 ms in the independent noise case, corresponding to $t_{\text{shift}}=200\;\text{ms}$, the burst times are completely scrambled.

#### Structured connectivity

We assume throughout the majority of this study that synapses are distributed randomly, without reference to the particular classification problem that needs to be solved. For comparison, we also assessed the performance of models in which the solution was increasingly ‘hard-wired’ by the placement of synapses on specific sets of branches (Figures S3D and S3E). Guided by the results of Figure 4C, for the active model, when two features $X_{i}$ and $Y_{i}$ formed a preferred association, their excitatory synapses were placed on a common set of basal dendrites, and separate sets of apical dendrites. Conversely, when two features formed a non-preferred association, their synapses were placed on separate basal dendrites and common apical dendrites. This arrangement should allow the model to take maximal advantage of supralinear integration in basal dendrites and sublinear integration in apical dendrites to solve the task. For the passive model, for both basal and apical dendrites, we separated synapses representing features forming preferred associations and clustered synapses forming nonpreferred associations.

To implement the clustering we randomly selected two equally sized and possibly overlapping sets of basal branches (from a possible 16), and two equally sized and possibly overlapping sets of apical branches (from a possible 8). Synapses to be clustered within a dendritic domain were randomly distributed across the same set of branches, whereas synapses to be dispersed were distributed across different sets. We parameterized the degree of structured connectivity in terms of the overlap between the two selected branch sets within each domain (1 minus the fraction of shared branches). For completely unstructured connectivity (0 in the parameterization), the two sets selected within each domain were identical, each consisting of all 16 basal or 8 apical branches. In this condition, as in the simulations in the main text, synapse placement does not depend on the classification labels. For maximally structured connectivity (1 in the parameterization), the two sets selected within each domain were disjoint, consisting of 8 basal or 4 apical branches each, such that convergence of input on common dendrites was completely prescribed by the classification labels. At intermediate levels the two sets selected within each domain partially overlapped. For instance, at 0.5 in the parameterization, the two sets of basal dendrites consisted of 12 branches each, half of which were common to both. For each condition we trained models using the classification labels that were favored by the imposed connectivity (‘favored labels’), and also with the contingencies switched (‘reversed labels’).

#### Active intrinsic conductances

To assess the possible role of additional active dendritic conductances beyond NMDA receptors, we extended the model to include a low density of dendritic voltage-dependent ${\text{Na}}^{+}$ and ${\text{K}}^{+}$ channels, as well as axo-somatic and dendritic HCN channels (see Dynamics and variational equations). Dendritic ${\text{Na}}^{+}$ conductances are responsible for fast dendritic spikes observed in pyramidal neurons, and serve to sharpen stimulus tuning and action potential timing (Ariav et al., 2003; Smith et al., 2013). HCN conductances are responsible for depolarizing ${\text{I}}_{\text{h}}$ currents, regulating the coupling between apical and somatic compartments and counterbalancing the effects of dendritic filtering (Magee, 1999; Harnett et al., 2015). We chose parameters such that dendritic ${\text{Na}}^{+}$ spikes were reliably triggered by synaptic input and the HCN-dependent voltage sag response to hyperpolarizing input was consistent with experimental observations (Kalmbach et al., 2018). We did not seek to precisely reproduce the experimental data, but rather to capture these effects qualitatively in a regime of dendritic excitability beyond that of the main NMDA-dependent active model.

We initially proceeded as before by performing many simulations with random input to fit plasticity kernels, as in Figure 2B. However, we found that using the same spike-triggered average approach as for the main models, we could not account for a comparable amount of the variance in $\frac{\partial v_{\text{soma}}}{\partial w}$. Specifically, whereas the local approximation accounts for ∼90% of the variance in the active-NMDA model (Figure 2C), in the presence of dendritic ${\text{Na}}^{+}$ spikes this was reduced to ∼80% in basal dendrites and ∼30% in apical dendrites. As this discrepancy would make a comparison between models unbalanced, we instead performed simulations in which the plasticity rule of Equation 27 used values of $\frac{\partial v_{\text{soma}}}{\partial w}$ computed directly for each synapse via numerical integration of (Equation 13), (Equation 14), (Equation 15), (Equation 16), (Equation 17) (i.e., we did not apply the approximation of Equation 26). We used the same sub-simulation procedure as described in Plasticity kernels, with the difference that the equations were integrated directly for synapses receiving multiple inputs, rather than copying synapses to solve separately for each individual synaptic activation. We applied this strategy in simulations with the original passive and active models, an active model with additional HCN conductances, an active model with additional dendritic ${\text{Na}}^{+}$ and ${\text{K}}^{+}$ conductances, and an active model with additional HCN, ${\text{Na}}^{+}$ and ${\text{K}}^{+}$ conductances. All models were trained and tested on the rate-coded 2×2 association task of Figure 3. To test whether the models with additional dendritic mechanisms obeyed the same rules of integration, we simulated and analyzed the subthreshold response to input features as in Figure 3D, setting the fast axo-somatic ${\text{Na}}^{+}$ and ${\text{K}}^{+}$ conductances to zero, but leaving all other conductances intact.

### Quantification and statistical analysis

Two-tailed Wilcoxon signed-rank tests were used to compare the performance and properties of models that were trained and tested on the same input patterns. Sample sizes and p values are provided in the figures and figure legends.

## Data Availability

This paper utilizes publicly available data from the Allen Cell Types Database. The accession numbers are listed in the key resources table. Simulation code is available at https://github.com/babicknell/Dendrites; https://doi.org/10.5281/zenodo.5524314

## Supplemental information

Document S1. Figures S1–S7

Document S2. Article plus supplemental information

## References

[bib1] Abrahamsson T., Cathala L., Matsui K., Shigemoto R., Digregorio D.A. (2012). Thin dendrites of cerebellar interneurons confer sublinear synaptic integration and a gradient of short-term plasticity. Neuron.

[bib2] Allen Institute for Brain Science (2015). http://celltypes.brain-map.org.

[bib3] Angelo K., London M., Christensen S.R., Häusser M. (2007). Local and global effects of Ih distribution in dendrites of mammalian neurons. J. Neurosci..

[bib4] Archie K.A., Mel B.W. (2000). A model for intradendritic computation of binocular disparity. Nat. Neurosci..

[bib5] Ariav G., Polsky A., Schiller J. (2003). Submillisecond precision of the input-output transformation function mediated by fast sodium dendritic spikes in basal dendrites of CA1 pyramidal neurons. J. Neurosci..

[bib6] Beniaguev D., Segev I., London M. (2021). Single cortical neurons as deep artificial neural networks. Neuron.

[bib7] Bhalla U.S. (2017). Synaptic input sequence discrimination on behavioral timescales mediated by reaction-diffusion chemistry in dendrites. eLife.

[bib8] Bittner K.C., Grienberger C., Vaidya S.P., Milstein A.D., Macklin J.J., Suh J., Tonegawa S., Magee J.C. (2015). Conjunctive input processing drives feature selectivity in hippocampal CA1 neurons. Nat. Neurosci..

[bib9] Bono J., Clopath C. (2017). Modeling somatic and dendritic spike mediated plasticity at the single neuron and network level. Nat. Commun..

[bib10] Branco T., Häusser M. (2011). Synaptic integration gradients in single cortical pyramidal cell dendrites. Neuron.

[bib11] Branco T., Clark B.A., Häusser M. (2010). Dendritic discrimination of temporal input sequences in cortical neurons. Science.

[bib12] Brunel N., Hakim V., Isope P., Nadal J.P., Barbour B. (2004). Optimal information storage and the distribution of synaptic weights: perceptron versus Purkinje cell. Neuron.

[bib13] Cash S., Yuste R. (1999). Linear summation of excitatory inputs by CA1 pyramidal neurons. Neuron.

[bib14] Cazé R.D., Humphries M., Gutkin B. (2013). Passive dendrites enable single neurons to compute linearly non-separable functions. PLoS Comput. Biol..

[bib15] Clopath C., Büsing L., Vasilaki E., Gerstner W. (2010). Connectivity reflects coding: a model of voltage-based STDP with homeostasis. Nat. Neurosci..

[bib16] Dayan P., Abbott L.F. (2001).

[bib17] Doron M., Chindemi G., Muller E., Markram H., Segev I. (2017). Timed synaptic inhibition shapes NMDA spikes, influencing local dendritic processing and global I/O properties of cortical neurons. Cell Rep..

[bib18] Doya K., Selverston A.I., Rowat P.F. (1994). A Hodgkin-Huxley type neuron model that learns slow non-spike oscillation. Adv. Neural Inf. Process. Syst..

[bib19] Ebner C., Clopath C., Jedlicka P., Cuntz H. (2019). Unifying long-term plasticity rules for excitatory synapses by modeling dendrites of cortical pyramidal neurons. Cell Rep..

[bib20] Gidon A., Segev I. (2012). Principles governing the operation of synaptic inhibition in dendrites. Neuron.

[bib21] Gidon A., Zolnik T.A., Fidzinski P., Bolduan F., Papoutsi A., Poirazi P., Holtkamp M., Vida I., Larkum M.E. (2020). Dendritic action potentials and computation in human layer 2/3 cortical neurons. Science.

[bib22] Goetz L., Roth A., Häusser M. (2021). Active dendrites enable strong but sparse inputs to determine orientation selectivity. Proc. Natl. Acad. Sci. U S A.

[bib23] Guerguiev J., Lillicrap T.P., Richards B.A. (2017). Towards deep learning with segregated dendrites. eLife.

[bib24] Gütig R. (2014). To spike, or when to spike?. Curr. Opin. Neurobiol..

[bib25] Gütig R., Sompolinsky H. (2006). The tempotron: a neuron that learns spike timing-based decisions. Nat. Neurosci..

[bib26] Gütig R., Sompolinsky H. (2009). Time-warp-invariant neuronal processing. PLoS Biol..

[bib27] Harnett M.T., Magee J.C., Williams S.R. (2015). Distribution and function of HCN channels in the apical dendritic tuft of neocortical pyramidal neurons. J. Neurosci..

[bib28] Harris C.R., Millman K.J., van der Walt S.J., Gommers R., Virtanen P., Cournapeau D., Wieser E., Taylor J., Berg S., Smith N.J. (2020). Array programming with NumPy. Nature.

[bib29] Häusser M., Mel B. (2003). Dendrites: bug or feature?. Curr. Opin. Neurobiol..

[bib30] Hawkins J., Ahmad S. (2016). Why neurons have thousands of synapses, a theory of sequence memory in neocortex. Front. Neural Circuits.

[bib31] Hay E., Hill S., Schürmann F., Markram H., Segev I. (2011). Models of neocortical layer 5b pyramidal cells capturing a wide range of dendritic and perisomatic active properties. PLoS Comput. Biol..

[bib32] Hines M. (1984). Efficient computation of branched nerve equations. Int. J. Biomed. Comput..

[bib33] Hines M., Carnevale N. (2006).

[bib34] Holmes W.R., Rall W. (1992). Estimating the electrotonic structure of neurons with compartmental models. J. Neurophysiol..

[bib35] Jadi M.P., Behabadi B.F., Poleg-Polsky A., Schiller J., Mel B.W. (2014). An augmented two-layer model captures nonlinear analog spatial integration effects in pyramidal neuron dendrites. Proc. IEEE Inst. Electr. Electron. Eng..

[bib36] Jahr C.E., Stevens C.F. (1990). Voltage dependence of NMDA-activated macroscopic conductances predicted by single-channel kinetics. J. Neurosci..

[bib38] Jones I.S., Kording K.P. (2021). Might a single neuron solve interesting machine learning problems through successive computations on its dendritic tree?. Neural Comput..

[bib37] Jones I.S., Kording K.P. (2021). Do biological constraints impair dendritic computation?. Neuroscience.

[bib39] Kalmbach B.E., Buchin A., Long B., Close J., Nandi A., Miller J.A., Bakken T.E., Hodge R.D., Chong P., de Frates R. (2018). h-Channels contribute to divergent intrinsic membrane properties of supragranular pyramidal neurons in human versus mouse cerebral cortex. Neuron.

[bib40] Kastellakis G., Cai D.J., Mednick S.C., Silva A.J., Poirazi P. (2015). Synaptic clustering within dendrites: an emerging theory of memory formation. Prog. Neurobiol..

[bib41] Kastellakis G., Silva A.J., Poirazi P. (2016). Linking memories across time via neuronal and dendritic overlaps in model neurons with active dendrites. Cell Rep..

[bib42] Kerlin A., Mohar B., Flickinger D., MacLennan B.J., Dean M.B., Davis C., Spruston N., Svoboda K. (2019). Functional clustering of dendritic activity during decision-making. eLife.

[bib43] Kitamura K., Judkewitz B., Kano M., Denk W., Häusser M. (2008). Targeted patch-clamp recordings and single-cell electroporation of unlabeled neurons in vivo. Nat. Methods.

[bib44] Koch C. (2002).

[bib45] Kole M.H., Hallermann S., Stuart G.J. (2006). Single Ih channels in pyramidal neuron dendrites: properties, distribution, and impact on action potential output. J. Neurosci..

[bib46] Körding K.P., König P. (2001). Supervised and unsupervised learning with two sites of synaptic integration. J. Comput. Neurosci..

[bib47] Krahe R., Gabbiani F. (2004). Burst firing in sensory systems. Nat. Rev. Neurosci..

[bib48] Lam S.K., Pitrou A., Seibert S. (2015). Proceedings of the Second Workshop on the LLVM Compiler Infrastructure in HPC.

[bib49] Larkum M.E., Zhu J.J., Sakmann B. (1999). A new cellular mechanism for coupling inputs arriving at different cortical layers. Nature.

[bib50] Legenstein R., Maass W. (2011). Branch-specific plasticity enables self-organization of nonlinear computation in single neurons. J. Neurosci..

[bib51] Legenstein R., Naeger C., Maass W. (2005). What can a neuron learn with spike-timing-dependent plasticity?. Neural Comput..

[bib52] Leventhal A.G., Thompson K.G., Liu D., Zhou Y., Ault S.J. (1995). Concomitant sensitivity to orientation, direction, and color of cells in layers 2, 3, and 4 of monkey striate cortex. J. Neurosci..

[bib53] Lisman J.E. (1997). Bursts as a unit of neural information: making unreliable synapses reliable. Trends Neurosci..

[bib54] London M., Häusser M. (2005). Dendritic computation. Annu. Rev. Neurosci..

[bib55] Losonczy A., Makara J.K., Magee J.C. (2008). Compartmentalized dendritic plasticity and input feature storage in neurons. Nature.

[bib56] Magee J.C. (1999). Dendritic lh normalizes temporal summation in hippocampal CA1 neurons. Nat. Neurosci..

[bib57] Major G., Larkum M.E., Schiller J. (2013). Active properties of neocortical pyramidal neuron dendrites. Annu. Rev. Neurosci..

[bib58] Margrie T.W., Meyer A.H., Caputi A., Monyer H., Hasan M.T., Schaefer A.T., Denk W., Brecht M. (2003). Targeted whole-cell recordings in the mammalian brain in vivo. Neuron.

[bib59] Mel B.W. (1992). The clusteron: toward a simple abstraction for a complex neuron. Adv. Neural Inf. Proc. Syst..

[bib60] Mel B.W. (1992). NMDA-based pattern discrimination in a modeled cortical neuron. Neural Comput..

[bib61] Minsky M., Papert S.A. (2017).

[bib62] Moldwin T., Segev I. (2020). Perceptron learning and classification in a modeled cortical pyramidal cell. Front. Comput. Neurosci..

[bib63] Moldwin T., Kalmenson M., Segev I. (2021). The gradient clusteron: a model neuron that learns to solve classification tasks via dendritic nonlinearities, structural plasticity, and gradient descent. PLoS Comput. Biol..

[bib64] Naud R., Sprekeler H. (2018). Sparse bursts optimize information transmission in a multiplexed neural code. Proc. Natl. Acad. Sci. USA.

[bib65] Nevian T., Larkum M.E., Polsky A., Schiller J. (2007). Properties of basal dendrites of layer 5 pyramidal neurons: a direct patch-clamp recording study. Nat. Neurosci..

[bib66] O’Donnell C., Sejnowski T.J. (2014). Selective memory generalization by spatial patterning of protein synthesis. Neuron.

[bib67] Palmer L.M., Shai A.S., Reeve J.E., Anderson H.L., Paulsen O., Larkum M.E. (2014). NMDA spikes enhance action potential generation during sensory input. Nat. Neurosci..

[bib68] Payeur A., Guerguiev J., Zenke F., Richards B.A., Naud R. (2021). Burst-dependent synaptic plasticity can coordinate learning in hierarchical circuits. Nat. Neurosci..

[bib69] Poirazi P., Mel B.W. (2001). Impact of active dendrites and structural plasticity on the memory capacity of neural tissue. Neuron.

[bib70] Poirazi P., Papoutsi A. (2020). Illuminating dendritic function with computational models. Nat. Rev. Neurosci..

[bib71] Poirazi P., Brannon T., Mel B.W. (2003). Arithmetic of subthreshold synaptic summation in a model CA1 pyramidal cell. Neuron.

[bib72] Poirazi P., Brannon T., Mel B.W. (2003). Pyramidal neuron as two-layer neural network. Neuron.

[bib73] Poleg-Polsky A. (2015). Effects of neural morphology and input distribution on synaptic processing by global and focal nmda-spikes. PLoS ONE.

[bib74] Polsky A., Mel B.W., Schiller J. (2004). Computational subunits in thin dendrites of pyramidal cells. Nat. Neurosci..

[bib75] Pospischil M., Toledo-Rodriguez M., Monier C., Piwkowska Z., Bal T., Frégnac Y., Markram H., Destexhe A. (2008). Minimal Hodgkin-Huxley type models for different classes of cortical and thalamic neurons. Biol. Cybern..

[bib76] Rall W., Reiss R.F. (1964).

[bib77] Rall W. (1967). Distinguishing theoretical synaptic potentials computed for different soma-dendritic distributions of synaptic input. J. Neurophysiol..

[bib78] Rosenblatt F. (1958). The perceptron: a probabilistic model for information storage and organization in the brain. Psychol. Rev..

[bib104] Sacramento J., Costa R.P., Bengio Y., Senn W. (2018). Dendritic cortical microcircuits approximate the backpropagation algorithm. Adv. Neural Inf. Process. Syst..

[bib79] Schiess M., Urbanczik R., Senn W. (2016). Somato-dendritic synaptic plasticity and error-backpropagation in active dendrites. PLoS Comput. Biol..

[bib80] Schiller J., Major G., Koester H.J., Schiller Y. (2000). NMDA spikes in basal dendrites of cortical pyramidal neurons. Nature.

[bib81] Seol G.H., Ziburkus J., Huang S., Song L., Kim I.T., Takamiya K., Huganir R.L., Lee H.K., Kirkwood A. (2007). Neuromodulators control the polarity of spike-timing-dependent synaptic plasticity. Neuron.

[bib82] Sezener E., Grabska-Barwinska A., Kostadinov D., Beau M., Krishnagopal S., Budden D., Hutter M., Veness J., Botvinick M., Clopath C. (2021). A rapid and efficient learning rule for biological neural circuits. bioRxiv.

[bib83] Silver R.A. (2010). Neuronal arithmetic. Nat. Rev. Neurosci..

[bib84] Sjöström P.J., Rancz E.A., Roth A., Häusser M. (2008). Dendritic excitability and synaptic plasticity. Physiol. Rev..

[bib85] Smith S.L., Smith I.T., Branco T., Häusser M. (2013). Dendritic spikes enhance stimulus selectivity in cortical neurons in vivo. Nature.

[bib86] Spruston N. (2008). Pyramidal neurons: dendritic structure and synaptic integration. Nat. Rev. Neurosci..

[bib87] Steuber V., Mittmann W., Hoebeek F.E., Silver R.A., De Zeeuw C.I., Häusser M., De Schutter E. (2007). Cerebellar LTD and pattern recognition by Purkinje cells. Neuron.

[bib88] Stuart G.J., Sakmann B. (1994). Active propagation of somatic action potentials into neocortical pyramidal cell dendrites. Nature.

[bib89] Stuart G., Schiller J., Sakmann B. (1997). Action potential initiation and propagation in rat neocortical pyramidal neurons. J. Physiol..

[bib90] Tran-Van-Minh A., Cazé R.D., Abrahamsson T., Cathala L., Gutkin B.S., DiGregorio D.A. (2015). Contribution of sublinear and supralinear dendritic integration to neuronal computations. Front. Cell. Neurosci..

[bib91] Tzilivaki A., Kastellakis G., Poirazi P. (2019). Challenging the point neuron dogma: FS basket cells as 2-stage nonlinear integrators. Nat. Commun..

[bib92] Ujfalussy B.B., Makara J.K. (2020). Impact of functional synapse clusters on neuronal response selectivity. Nat. Commun..

[bib93] Ujfalussy B.B., Makara J.K., Branco T., Lengyel M. (2015). Dendritic nonlinearities are tuned for efficient spike-based computations in cortical circuits. eLife.

[bib94] Ujfalussy B.B., Makara J.K., Lengyel M., Branco T. (2018). Global and multiplexed dendritic computations under in vivo-like conditions. Neuron.

[bib95] Urbanczik R., Senn W. (2009). A gradient learning rule for the tempotron. Neural Comput..

[bib96] Urbanczik R., Senn W. (2014). Learning by the dendritic prediction of somatic spiking. Neuron.

[bib97] Van Rossum G., Drake F.L. (2009).

[bib98] Vervaeke K., Lőrincz A., Nusser Z., Silver R.A. (2012). Gap junctions compensate for sublinear dendritic integration in an inhibitory network. Science.

[bib99] Vetter P., Roth A., Häusser M. (2001). Propagation of action potentials in dendrites depends on dendritic morphology. J. Neurophysiol..

[bib100] Williams S.R., Stuart G.J. (2002). Dependence of EPSP efficacy on synapse location in neocortical pyramidal neurons. Science.

[bib101] Wu X.E., Mel B.W. (2009). Capacity-enhancing synaptic learning rules in a medial temporal lobe online learning model. Neuron.

[bib102] Zador A., Claiborne B., Brown T. (1991). Nonlinear pattern separation in single hippocampal neurons with active dendritic membrane. Adv. Neural Inf. Process. Syst..

[bib103] Zucker R.S., Regehr W.G. (2002). Short-term synaptic plasticity. Annu. Rev. Physiol..

