# Supplementary material for: A synaptic learning rule for exploiting nonlinear dendritic computation
Source: Neuron. 2021 Dec 15;109(24):4001–4017.e10. doi: 10.1016/j.neuron.2021.09.044 (PMC8691952; doi:10.1016/j.neuron.2021.09.044)
Supplement: Document S1. Figures S1–S7 [file mmc1.pdf]

Neuron, Volume *109*

Supplemental information

A synaptic learning rule for exploiting nonlinear dendritic computation

Brendan A. Bicknell and Michael Häusser

Supplemental Figures

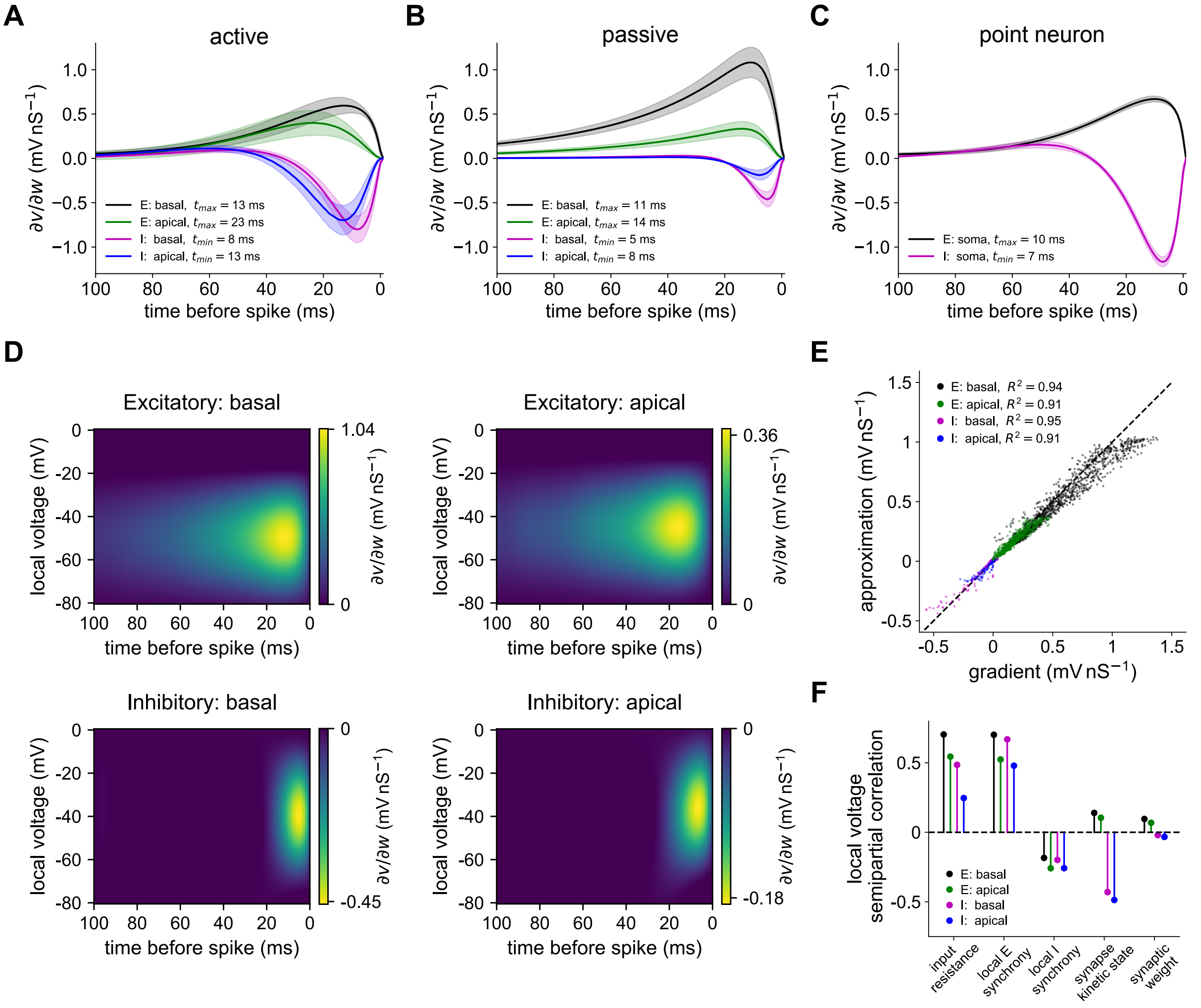

Figure S1: **Synaptic integration in active, passive and point neuron models. Related to Figure 2.** (A) Spike-triggered averages of synaptic influence ($\frac{\partial v_{\text{soma}}}{\partial w}$) in the active model for excitatory and inhibitory synapses in basal and apical dendrites. (B) Spike-triggered average $\frac{\partial v_{\text{soma}}}{\partial w}$ in the passive model. Note that inhibitory influence is markedly reduced compared to the active model. In this case brief inhibitory inputs cannot veto long-lived ohmic NMDA currents, as they can with the voltage-dependent NMDA receptors (Doron et al. 2017). (C) Spike-triggered average $\frac{\partial v_{\text{soma}}}{\partial w}$ in the point neuron model. All shaded areas are s.d. (D) Polynomial fits of two-dimensional spike-triggered averages for the passive model, to be used as plasticity kernels. (E) The approximations in panel D accurately predict the voltage gradients computed from numerical integration of equations (13)-(17) (fitted on 75% of the simulated data and tested on the remaining 25%). For visibility, scatter plot shows randomly sampled points from bins of 0.1 mV nS^{-1} width along the x-axis (up to 100 points per bin). R^2 values are computed from the correlation between actual and approximated values over all held-out data. (F) The voltage at a synapse at the time of somatic spikes in the passive model depends on multiple factors, allowing their implicit representation in the learning rule. Semipartial correlation was computed from a linear model fitted on 75% of the data and tested on the remaining 25%.

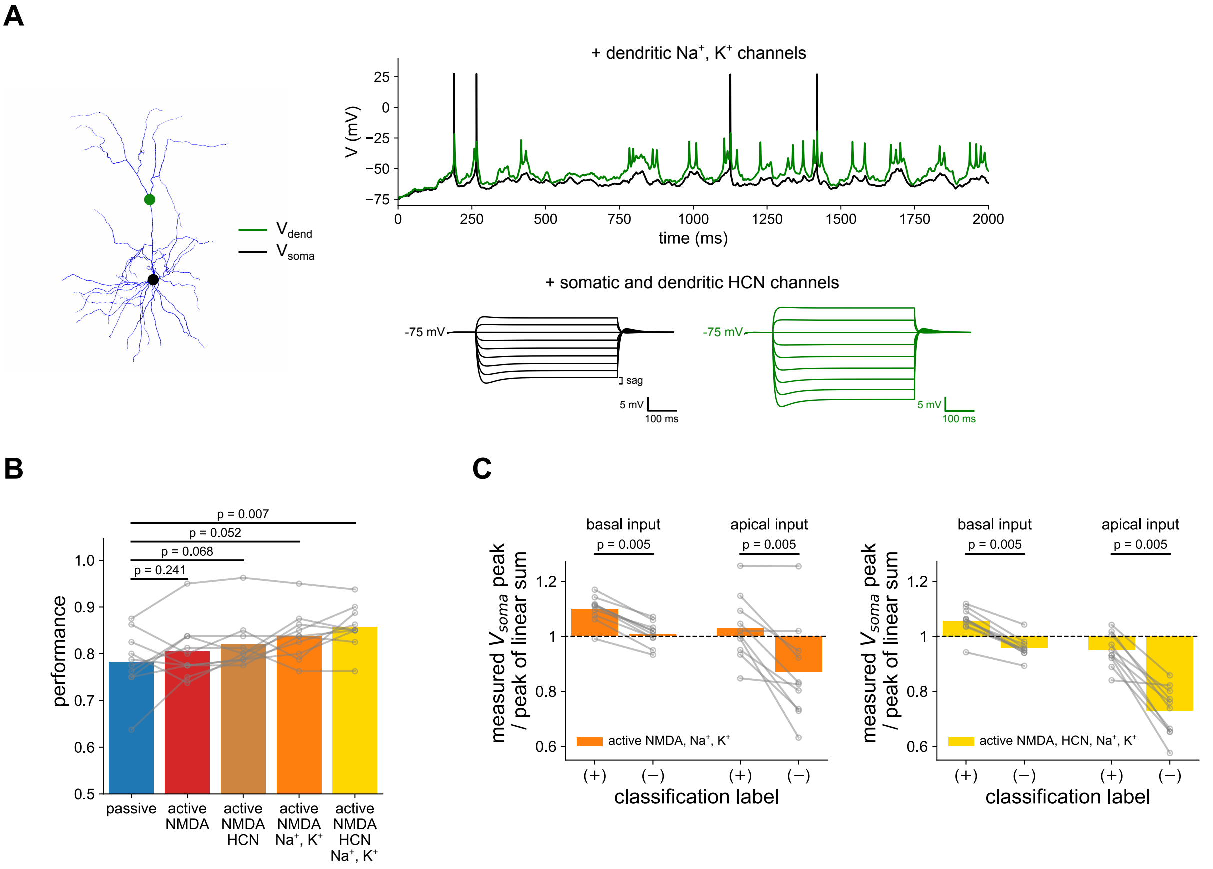

Figure S2: **Simulations with active intrinsic dendritic conductances. Related to Figure 3.** (A) Left: Pyramidal cell morphology showing sites of somatic (black) and dendritic (green) recording and current injection for the simulations shown on the right. Top right: Example simulation of a model stimulated with Poisson input. Dendritic Na^+ (fast transient) and K^+ (delayed rectifier and slow persistent) conductances have been added to the active model used in the simulations in the main text. Fast Na^+ spikes are generated in the dendrites, riding on envelopes of NMDA-dependent depolarization that remain visible at the soma. Bottom right: Simulation of a model with dendritic Na^+ and K^+ conductances, and somatic and dendritic HCN conductances. Shown is the response to somatic and dendritic current injection in 25 pA steps from −150 to 50 pA. Current was injected at the locations indicated on the left, and the voltage recorded at the same site. Parameter values were chosen to produce a voltage sag (difference between steady-state and minimum voltage) of 1−2 mV, consistent with experimental observations. (B) Performance of a hierarchy of models trained in the rate-coded 2×2 association task. The x-axis lists the active mechanisms present in the dendritic tree of each model. For these simulations the gradient term $\frac{\partial v_{\text{soma}}}{\partial w}$ used in the learning rule was numerically integrated using equations (13)-(17), rather than approximated using local plasticity kernels. (C) Analysis of supra/sublinear integration with active intrinsic conductances. The integrative strategy is consistent with the active-NMDA model used throughout the main text.

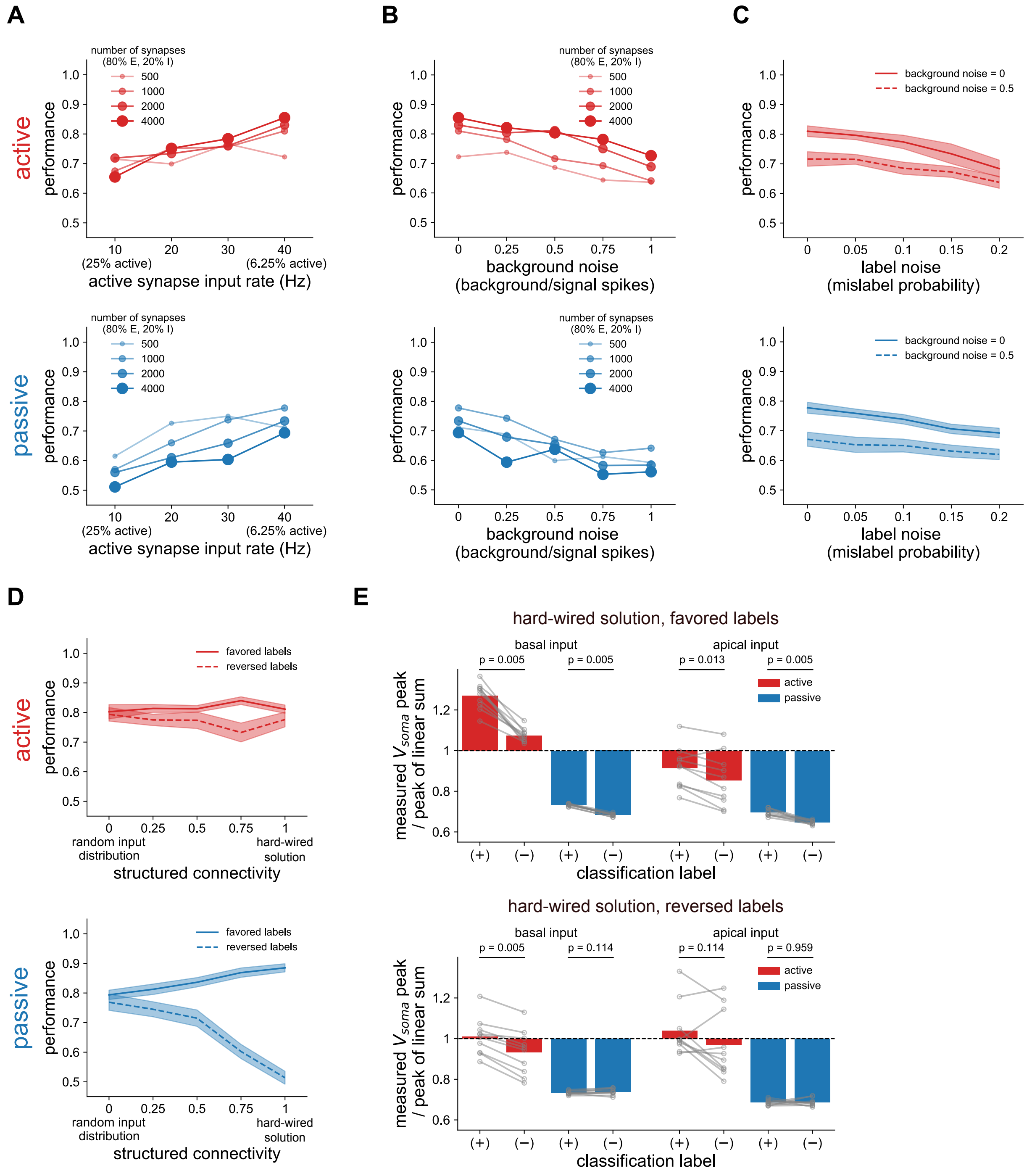

Figure S3: **Noise robustness and dependence on structured connectivity. Related to Figures 3 and 4.** Results of additional simulations of the active (red) and passive (blue) models on the rate-coded 2×2 task under alternative noise and connectivity assumptions. Models were trained and tested on 10 random instantiations of the task. Performance is quantified as the fraction of correct classifications from 20 presentations of each input pattern after training. In panels A and B, the size of the marker corresponds to the total number of synapses used ($N_{\text{syn}} = 1000$ for all simulations in the main text), and errors bars are omitted for visual clarity. (A) Robustness to spike count variability. The rates of input to active synapses were varied while keeping the presynaptic population rate constant. At the level of an individual synapse, lower input rates result in a larger spike count standard deviation relative to the mean.

(B) Robustness to background synaptic activity. The level of background noise was varied by adding a constant λ_0 to the input rate of all synapses. At a noise level of 1, $\lambda_0 = 2.5\,\text{Hz}$ and there are as many background spikes in the presynaptic population as there are stimulus-dependent spikes. (C) Robustness to label noise. The reliability of the supervisory teaching signal was varied during training by randomly switching the classification label on individual trials. Shaded area is s.e.m. (D) Synapse placement was varied from the random input distribution used in simulations in the main text, to structured configurations in which the solution to the classification task is increasingly 'hard-wired' to reinforce the optimal patterns of functional clustering shown in Figure 4. Models were trained to perform the classification favored by the input wiring, as well as the classification with unfavorable, reversed labels. Shaded area is s.e.m. (E) Analysis of supra/sublinear integration with structured connectivity. Subthreshold potentials were simulated (as in Figure 3D) in models trained in the maximally hard-wired condition. For favored labels, by construction, the computational strategy is the same as that learned under a random input distribution. For reversed labels, while the passive model fails to solve the task, the more flexible active model learns to perform the classification through sublinear processing in basal dendrites. Bars denote means (averaged over 20 presentations of each association, then over label types), p-values are from two-tailed Wilcoxon signed-rank tests between groups for n=10 independent replications.

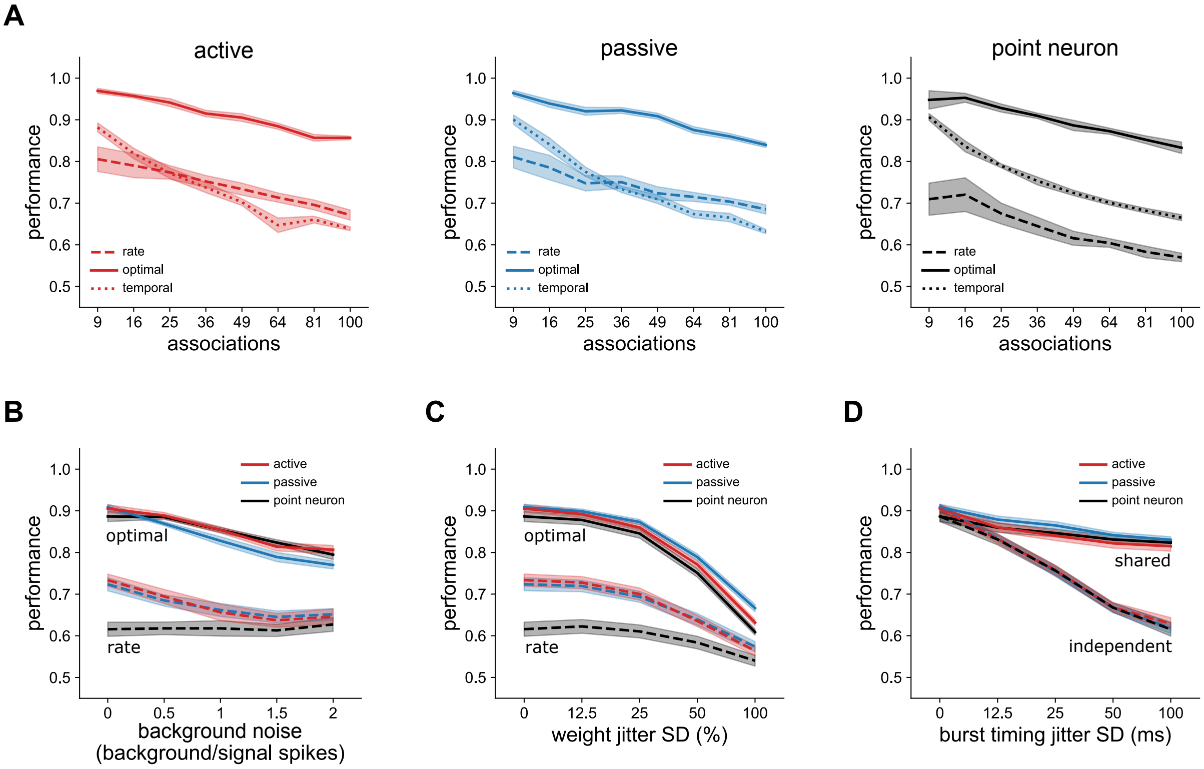

Figure S4: **Noise robustness and classification capacity with temporally coded input. Related to Figure 5.** (A) Performance of the active (red), passive (blue) and point neuron (black) models for increasing numbers of associations. Each value on the x-axis corresponds to an $n \times n$ feature-binding task with randomly assigned classification labels. Models were trained under the three input regimes shown in Figure 5: rate (dashed lines), optimal (solid lines), temporal (dotted line). Shaded area is s.e.m. from 10 random instantiations of the task. (B) Robustness to background synaptic activity on the 7×7 association task in the rate-coded and optimal input regimes. The level of background noise was varied by adding a constant λ_0 to the input rate of all synapses. At a noise level of 1, $\lambda_0 = 2.5\,\text{Hz}$ and there are as many background spikes in the presynaptic population as there are stimulus-dependent spikes. (C) Robustness to synaptic weight jitter on the 7×7 association task in the rate-coded and optimal input regimes. Trained models were tested on 20 presentations of each input pattern with all synaptic weights scaled by independent multiplicative noise terms. (D) Robustness to burst-timing jitter on the 7×7 association task in the optimal input regime. Trained models were tested on 20 presentations of each input pattern with burst times perturbed with additive noise. In the 'shared' noise condition, all burst times were periodically shifted by the same amount, corresponding to a random shift in the phase of the temporal stimulus. In the 'independent' noise condition, all burst times were shifted independently – in this case a timing jitter SD of 100 ms corresponds to a complete loss of temporal information.

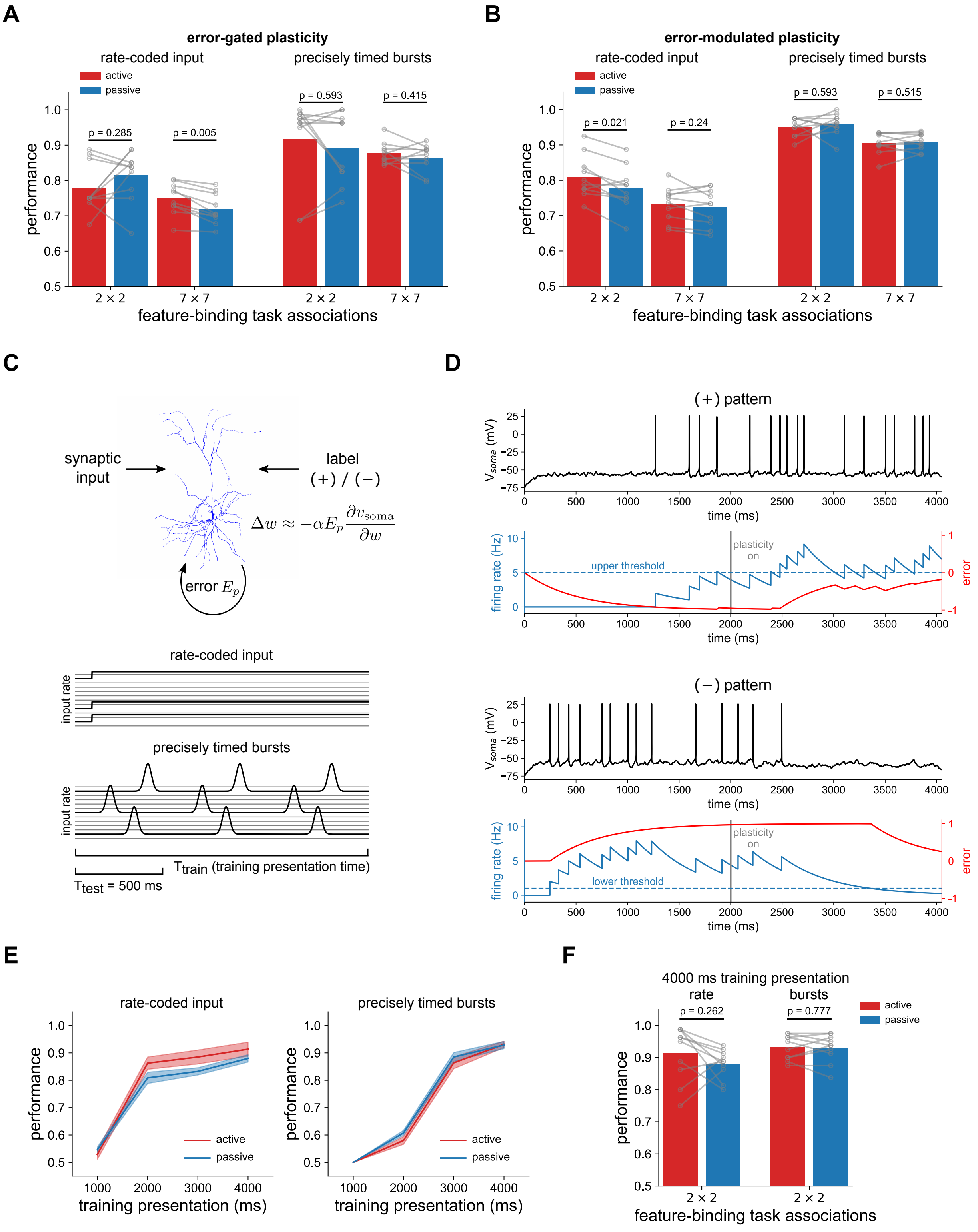

Figure S5: **Error-gated, error-modulated and online learning rules. Related to Figures 3, 5 and STAR Methods.** (A) Performance of models trained using an alternate version of the learning rule in which plasticity is gated on when the presentation of an input pattern results in an erroneous classification. In this case the running average error $\bar{E}_p$ does not scale the magnitude of weight updates. Separate learning rates were required to learn the 2×2 association task and the 7×7 association task (the latter fourfold lower). (B) Performance of

models trained with the learning rule used throughout the main text. The magnitudes of weight updates are modulated by the running average error (equation (27)). Data are replotted from the main text for comparison with panel A. With the adaptive dynamics of the error-modulated rule, performance is more consistent and a single learning rate suffices. (C) Schematic of an online implementation of the error-modulated learning rule. During training, input patterns are presented for a duration T_{train} (temporally coded input patterns are repeated periodically) while a supervisory signal provides a binary classification label. The classification error is computed through comparison of the output firing rate to a threshold, and fed back to the neuron to guide plasticity. After training, models are tested on pattern presentations of duration T_{test}. (D) Examples of the online learning dynamics for two input patterns. For the (+) pattern, the output firing rate (in this example, computed using averaging time constant $\tau_{\text{r}} = 500\,\text{ms}$) is initially below an upper threshold r_{U}, so the error decreases to $E_p = -1$. This causes an increase in somatic teaching current $I_{\text{teach}} = \beta|E_p|$, eliciting somatic spikes. At time $T_{\text{train}}/2$, plasticity is turned on and synaptic weights are updated with every somatic spike, increasing the firing rate above threshold. The error then returns to 0, decreasing the teaching current. Because the synaptic weights have been reinforced, the neuron now spikes without assistance. For the (−) pattern, an initially high firing rate drives the error to $E_p = 1$. When plasticity is turned on, weights are updated and the firing rate is pushed towards a lower threshold r_{L}. (E) Performance of models trained on the 2×2 association task using the online learning rule. For sufficiently large T_{train}, performance matches or exceeds that of the offline rule used in panel B. Shaded areas are s.e.m from 10 random instantiations of the task. (F) Detailed comparison of performance for $T_{\text{train}} = 4000\,\text{ms}$. Bars denote means, p-values are from two-tailed Wilcoxon signed-rank tests between groups for n=10 independent replications.

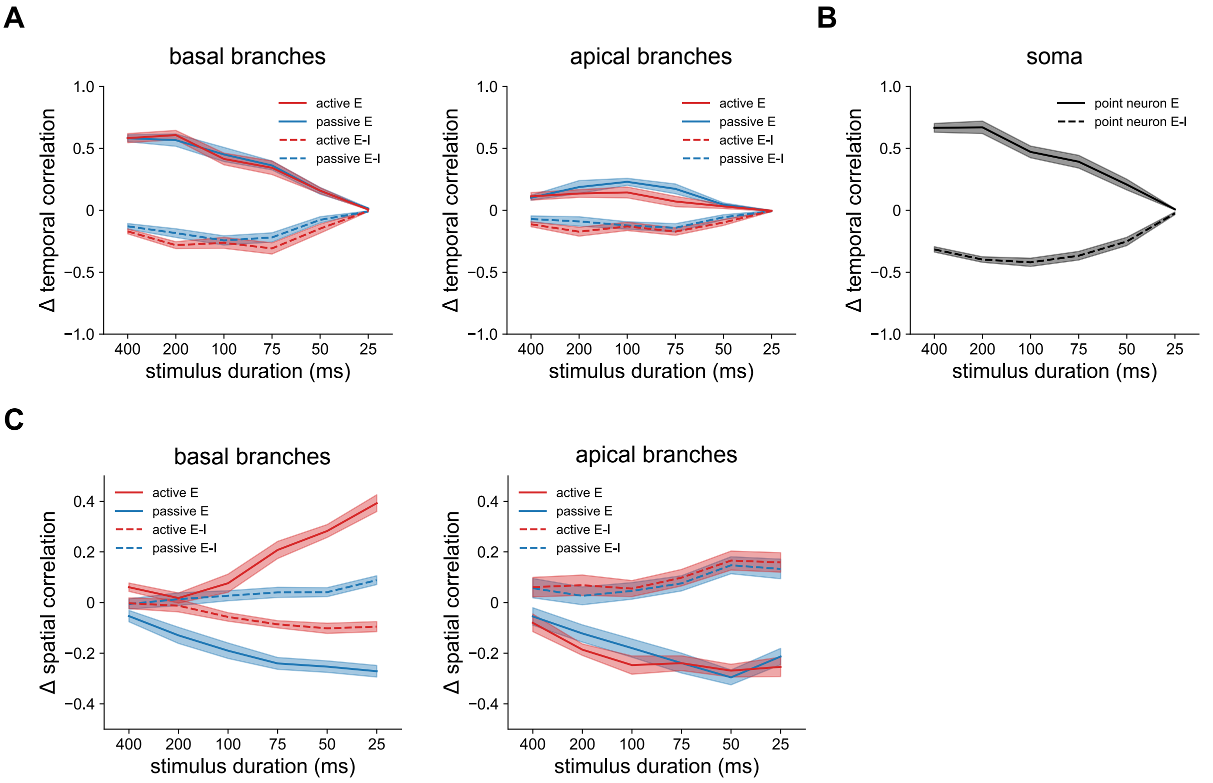

Figure S6: **Signatures of spatial and temporal processing as a function of stimulus duration. Related to Figure 7.** (A) Difference in average temporal input profile correlations between preferred and nonpreferred associations for the active (red) and passive (blue) models. E: excitatory (solid lines), E-I: excitatory-inhibitory (dashed lines). We refer to this collection of measurements in the main text as ρ_{T}. (B) As in panel A, but for the point neuron model. (C) As in panel A, but for the correlations between spatial input profiles (ρ_{S}). Shaded areas are s.e.m. from 10 replications.

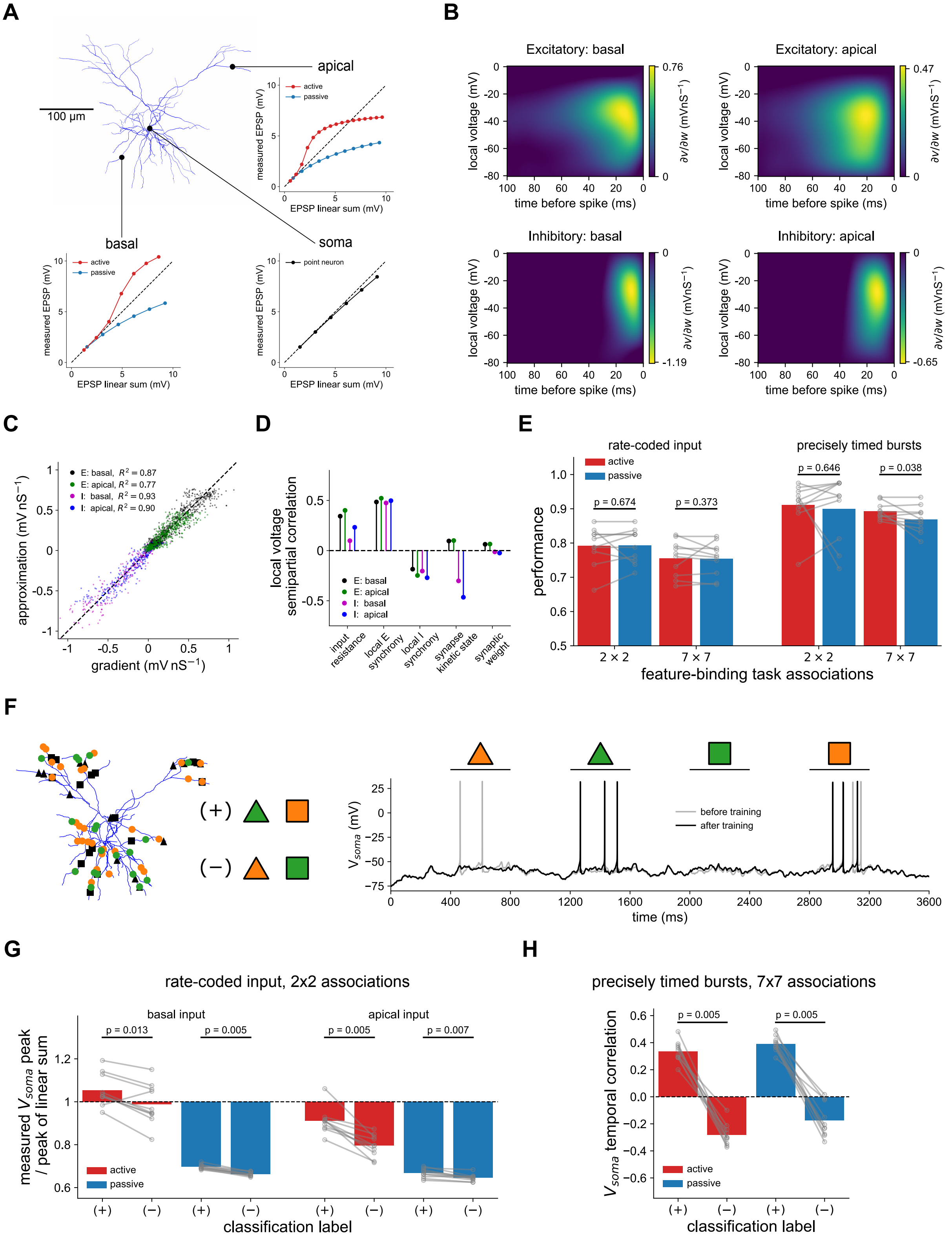

Figure S7: Replication with a different layer 2/3 pyramidal cell morphology. Related to Figures 2-6. (A) Simulated peak somatic response to increasing numbers of excitatory synaptic inputs at the indicated locations, compared to the peak of the linear sum of the same number of unitary EPSPs. Voltage-dependent NMDA receptors yield supralinear integration within dendritic branches (active model, red lines), whereas integration in a purely

passive model is sublinear (passive model, blue lines). Integration is approximately linear at the soma (point neuron model, black line). (B) Polynomial fits of somatic spike-triggered average $\frac{\partial v_{\text{soma}}}{\partial w}$ in the active model, to be used as plasticity kernels in the learning algorithm. (C) The approximations in panel B accurately predict the numerically integrated voltage gradients at the time of somatic spikes (fitted on 75% of the simulated data and tested on the remaining 25%). For visibility, scatter plot shows randomly sampled points from bins of $0.1\,\mathrm{mV\,nS^{-1}}$ width along the x-axis (up to 100 points per bin). R^2 values are computed from the correlation between actual and approximated values over all held-out data. (D) The voltage at a synapse at the time of somatic spikes depends on multiple factors, allowing their implicit representation in the learning rule. Semipartial correlation computed from a linear model fitted on 75% of the data and tested on the remaining 25%. (E) Performance on the feature binding task with rate-coded input (left bars) or precisely timed bursts (right bars). (F) Example simulation of model trained on the rate-coded 2×2 association task. Left: Synapse locations and classification labels for four 'color-shape' associations. Right: Somatic voltage before (gray) and after (black) training in response to the four associations presented in turn, interspersed with background noise. (G) Analysis of supra/sublinear integration for models trained with rate-coded input. (H) Analysis of temporal integration for models trained with precisely timed bursts. Bars denote means (averaged over replications for each association, then over label types), p-values are from two-tailed Wilcoxon signed-rank tests between groups for n=10 independent replications.
